# Claudin‐12‐Expressing Fibroblasts Facilitate Metastatic Spread of Pancreatic Cancer Through Physical Interactions

**DOI:** 10.1002/mco2.71114

**Published:** 2026-10-31

**Authors:** Qingcui Li, Xiangjing Miao, Yuanyuan Yang, Ruoyang Chen, Qingqing Fang, Xixi Duan, Xiaohan Lou, Xiaohan Yao, Lijing Zhang, Rainer Glauben, Zhihai Qin, Chen Ni

**Affiliations:** ^1^ Medical Research Center, Henan China‐Germany International Joint Laboratory of Tumor Immune Microenvironment and Disease the First Affiliated Hospital of Zhengzhou University Zhengzhou Henan China; ^2^ Key Laboratory of Glyco‐drug Research of Zhejiang Province, School of Pharmaceutical Science and Technology, Hangzhou Institute for Advanced Study University of Chinese Academy of Sciences Hangzhou Zhejiang China; ^3^ Department of Gastroenterology Infectious Diseases and Rheumatology Charité—Universitätsmedizin Berlin Corporate Member of Freie Universität Berlin and Humboldt‐Universität zu Berlin Berlin Germany

**Keywords:** cancer‐associated fibroblast, Claudin‐12, homophilic adhesion, metastasis, pancreatic cancer

## Abstract

Claudins (CLDNs), as major components of tight junctions in epithelial cells, are emerging as attractive non‐mutant targets for controlling the growth and metastasis of various cancers. However, the mechanisms by which CLDNs modulate tumor progression remain largely unclear. Here, we unexpectedly found that CLDN12 is expressed in cancer‐associated fibroblasts (CAFs), which are generally not considered tight junction‐forming cells. CLDN12 expression in fibroblasts increased their motility and, importantly, promoted direct adhesion to pancreatic cancer cells through homophilic CLDN12–CLDN12 interactions. This CLDN12‐dependent physical interaction between fibroblasts and tumor cells markedly enhanced cancer cell invasion. Moreover, TGF‐β produced by cancer cells further induced the upregulation of CLDN12 expression in fibroblasts. In vivo, CLDN12‐positive fibroblasts facilitated distant metastasis of pancreatic cancer to liver and diaphragm. Targeting CLDN12 with the synthetic peptide C12‐pm efficiently decreased the liver metastasis of pancreatic cancer. Taken together, these findings reveal a previously unrecognized role of CLDN12‐expressing fibroblasts in promoting pancreatic cancer metastasis through direct physical interactions with tumor cells and highlight CLDN12‐mediated interactions as a potential therapeutic target for metastatic pancreatic cancer.

## Graphical abstract

Tumor‐derived TGF‐β induces CLDN12 expression in cancer‐associated fibroblasts, enabling direct adhesion to pancreatic cancer cells through homophilic CLDN12 interactions, thereby promoting local invasion into adjacent tissues and metastasis to the liver; blockade with C12‐pm suppresses liver metastasis.

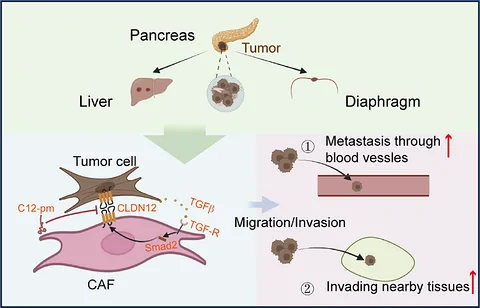

## 1 Introduction

Tight junctions on epithelial cells serve barrier and fence functions to regulate the paracellular passage of various molecules and to maintain cell polarity [1]. Claudins (CLDNs) are the major components of tight junctions and consist of ∼27 members [2]. CLDNs are tetraspan transmembrane proteins including two extracellular loops (ECLs), and their intercellular homotypic or heterotypic interactions form tight junction strands [2]. Loss of cell polarity and disruption of tight junctions have occurred in cancers probably due to their dedifferentiation [1, 3]. Claudins expressed on cancer cells or stromal cells have recently emerged as attractive non‐mutant therapeutic targets for controlling the growth and metastasis of cancers [1, 4, 5, 6]. However, the mechanisms by which CLDNs modulate tumor progression, especially by reshaping the tumor microenvironment, remain largely unclear.

As an atypical claudin family member, CLDN12 is widely expressed in epithelial cells, endothelial cells, pericytes, and Schwann cells [7, 8]. Although CLDN12 is not involved in the blood‐brain barrier tight‐junction function [9], the loss of CLDN12 affects the intestinal absorption of calcium [10] and the breakdown of the myelin barrier shielding the peripheral neurons [8]. The expression level of CLDN12 is altered in various cancer types [1, 11, 12, 13, 14]. Increased CLDN12 expression has been observed in lung squamous cell carcinoma tissues and is associated with lymphatic metastasis [14]. The expression of CLDN12 in pancreatic cancer cells can promote the proliferation and migration of these cells [15]. Our recent study showed that CLDN12 is expressed by myeloid‐derived suppressor cells (MDSCs) and facilitates their entry into tumors by CLDN12‐mediated adhesion between endothelial cells and MDSCs [16]. Nonetheless, the expression of CLDN12 by other stromal cells and its involvement in tumor growth or metastasis need to be examined.

Cancer‐associated fibroblasts (CAFs) are an important part of the tumor microenvironment and impact cancer invasion and metastasis, including pancreatic cancer. CAFs can indirectly communicate with cancer cells and promote cancer invasion and metastasis by secreting soluble factors, such as IL6 or VEGF [17, 18, 19, 20, 21]. Importantly, CAFs can also directly interact with cancer cells by cell–cell contact, leading to cancer cell invasion via adhesion molecules [22, 23, 24, 25], or transferring nutrients by gap junctions [26]. The expression of CLDNs (e.g. CLDN2 and 11) by CAFs has been observed [27, 28], and CLDN2 expression in CAFs of the invasive margin was associated with shorter progression‐free survival in colorectal cancer [28]. It has been reported that vascular heterogeneity of CLDNs guides organotropic metastasis of cancers [6]. However, the functions of CLDNs expressed by CAFs have not been clarified.

In this study, we identify an unexpected role of CLDN12 in CAFs. We demonstrate that CLDN12 mediates direct physical interactions between CAFs and pancreatic cancer cells, promoting cancer cell invasion and metastasis. Mechanistically, TGF‐β secreted by pancreatic cancer cells can upregulate the expression of CLDN12 in fibroblasts. In vivo, CLDN12‐expressing fibroblasts promoted the distant metastasis of pancreatic cancer to the liver and diaphragm. To explore the molecular basis of CLDN12‐mediated adhesion, we focused on the second extracellular loop (ECL2), a conserved functional domain of claudins involved in intercellular interactions. Disruption of CLDN12‐mediated cell–cell adhesion using a synthetic peptide targeting ECL2 (C12‐pm) efficiently suppressed liver metastasis. Collectively, CLDN12 mediates direct tumor‐CAF physical interactions, thereby promoting distant metastasis of pancreatic cancer by enhancing cancer cell invasion and providing a potential target for intervening in pancreatic cancer metastasis.

## 2 Results

### 2.1 CLDN12 Is Highly Expressed in CAFs of Pancreatic Cancer

To assess the expression of CLDN12 in fibroblasts, we first isolated primary embryonic fibroblasts (MEFs) from wild‐type (WT) mice and verified their identity by cellular immunofluorescence staining for α‐smooth muscle actin (α‐SMA). The fibroblast phenotype was further confirmed by Western blot analysis of canonical fibroblast markers, including COL1A1, S100A4, and α‐SMA (Figure 1A, B). The expression of *Cldn12* mRNA in MEF was determined by qRT‐PCR, with the endothelial cell line SVEC4‐10 included as a control based on our previous study showing CLDN12 expression in endothelial cells [16] (Figure 1C). To further determine whether CLDN12 is expressed in MEFs, Western blot analysis was performed. Based on the same previous study showing that CLDN12 is expressed in MDSCs, MSC2, a MDSC cell line, was included as a myeloid cell control, while RAW264.7 was included as an additional myeloid cell control (Figure 1D). Immortalized mouse embryonic fibroblasts (iMEFs) were stimulated with Pan02‐conditioned medium (CM) for 0, 12, 24, and 48 h. Western blot analysis and densitometric quantification showed that Pan02‐CM treatment progressively increased CLDN12 expression (Figure 1E), accompanied by elevated expression of the CAF activation marker α‐SMA (Figure S1A), indicating that tumor‐derived soluble factors promote CLDN12 upregulation during fibroblast activation. To evaluate CLDN12 expression in vivo, we examined pancreatic tissues from an orthotopic pancreatic KPC tumor model. In this model, normal fibroblasts (NFs) were identified in the non‐tumor adjacent pancreatic tissue surrounding the tumor, while CAFs were located within the tumor regions. Immunostaining showed that α‐SMA^+^ NFs in the peri‐tumoral normal tissue exhibited minimal CLDN12 expression, indicating a low basal level in quiescent fibroblasts. In contrast, α‐SMA^+^ CAFs within the tumor displayed strong CLDN12 positivity on the cell membrane, as indicated by the yellow signal resulting from the overlap of α‐SMA (red) and CLDN12 (green), suggesting that CLDN12 is specifically upregulated during fibroblast activation in the tumor context (Figure 1F). To further validate these findings, primary NFs were isolated from the normal adjacent pancreas and CAFs were isolated from the KPC pancreatic tumors (Figure 1G). qPCR analysis of *Acta2*, *Col1a1*, *Pdpn*, and *Fn1* confirmed their fibroblast identity (Figure 1H). Both NFs and CAFs expressed high levels of *Col1a1*, whereas *Acta2* expression was low in NFs and markedly elevated in CAFs. In addition, *Pdpn*, and *Fn1* were expressed at substantially higher levels in CAFs compared with NFs, consistent with their fibroblast identity. Notably, qRT‐PCR further revealed that *Cldn12* mRNA levels were significantly higher in CAFs than in NFs (Figure 1I), supporting our conclusion that CLDN12 is preferentially upregulated in tumor‐associated fibroblasts.

**FIGURE 1:**
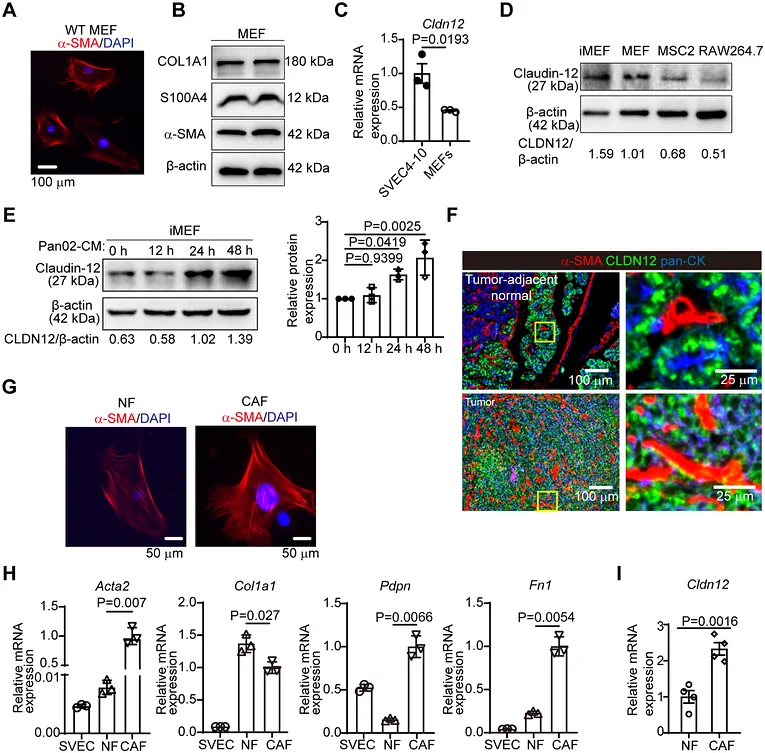
CLDN12 is expressed in fibroblasts. (A) Primary mouse embryonic fibroblasts (MEFs) were isolated from wild‐type (WT) C57BL/6 mice and stained with α‐SMA. Scale bar: 100 µm. (B) Expression of fibroblast‐associated marker proteins (COL1A1, S100A4, α‐SMA) in MEFs detected by western blotting. (C) qRT‐PCR analysis of *Cldn12* mRNA levels in MEFs, with the endothelial cell line SVEC4‐10 included as a control. *N* = 3 for each group, *t*‐test. (D) Western blot analysis of CLDN12 expression in MEFs, with MSC2 and RAW264.7 included as myeloid cell controls. Densitometric quantification of CLDN12 protein levels normalized to β‐actin is shown. (E) Western blot analysis of CLDN12 expression in iMEFs stimulated with Pan02‐conditioned medium (CM) for 0, 12, 24, and 48 h. CLDN12 protein levels in iMEFs were quantified by densitometric analysis, calculated as the CLDN12/β‐actin ratio, and normalized to the control group. *N* = 3 for each group, one‐way ANOVA. (F) Immunofluorescence staining of pancreatic tissues from the orthotopic pancreatic tumor model. Red: α‐SMA^+^ fibroblasts (NFs or CAFs); green: CLDN12; blue: pan‐CK. Scale bars: left, 100 µm; right, 25 µm. (G) Normal fibroblasts (NF) and cancer‐associated fibroblasts (CAFs) were stained with α‐SMA. Scale bar: 50 µm. (H) qPCR analysis of fibroblast marker gene expression in SVEC cells, primary NFs, and CAFs, SVEC refers to the SVEC4‐10 cell line. A broken y‐axis is used in the α‐SMA panel. *N* = 3 for each group, *t*‐test. (I) *Cldn12* mRNA expression in CAFs and NFs. *N* = 4 for each group, *t*‐test.

### 2.2 CLDN12 Enhances the Migration and Contraction of Fibroblasts

To evaluate the impact of CLDN12 on the function of fibroblasts, CLDN12 was knocked down in iMEF cells using lentiviral shRNA transduction (sh*Cldn12*, sh1‐3; control, sh‐nc) which bore a GFP‐sequence to facilitate positive selection by flow cytometry. The successful knockdown was verified by qRT‐PCR for all three shRNAs (sh1, sh2, and sh3), and sh2 was used in the following experiments if not indicated otherwise (Figure 2A). Efficient knockdown of CLDN12 was further confirmed at the protein level by western blotting (Figure 2B). The knockdown of CLDN12 in iMEF did not influence proliferation of iMEF (Figure 2C), but significantly decreased the migration of iMEF cells as examined by a Transwell assay (Figure 2D, E). A collagen gel shrinkage experiment was carried out to examine the effect of CLDN12 on the contractility of iMEF cells. TGF‐β, a known factor that can induce fibroblast contraction, was used as a positive control [29]. The loss of CLDN12 significantly decreased the collagen gel contractility in iMEF with or without TGF‐β‐stimulation (Figure 2F, G). When CLDN12 was knocked down in CAFs (Figure 2H, I), the migration of CAF^sh^ *^Cldn12^* was reduced compared to CAF^sh‐nc^ (Figure 2J, K). In addition, time‐lapse microscopy was applied to track the motion of fibroblasts in real‐time. The results showed that the motion of CAF^sh^ *^Cldn12^* was reduced compared to CAF^sh‐nc^ cells (Figure 2L, M). These results suggest that the expression of CLDN12 enhances migration and contraction of fibroblasts.

**FIGURE 2:**
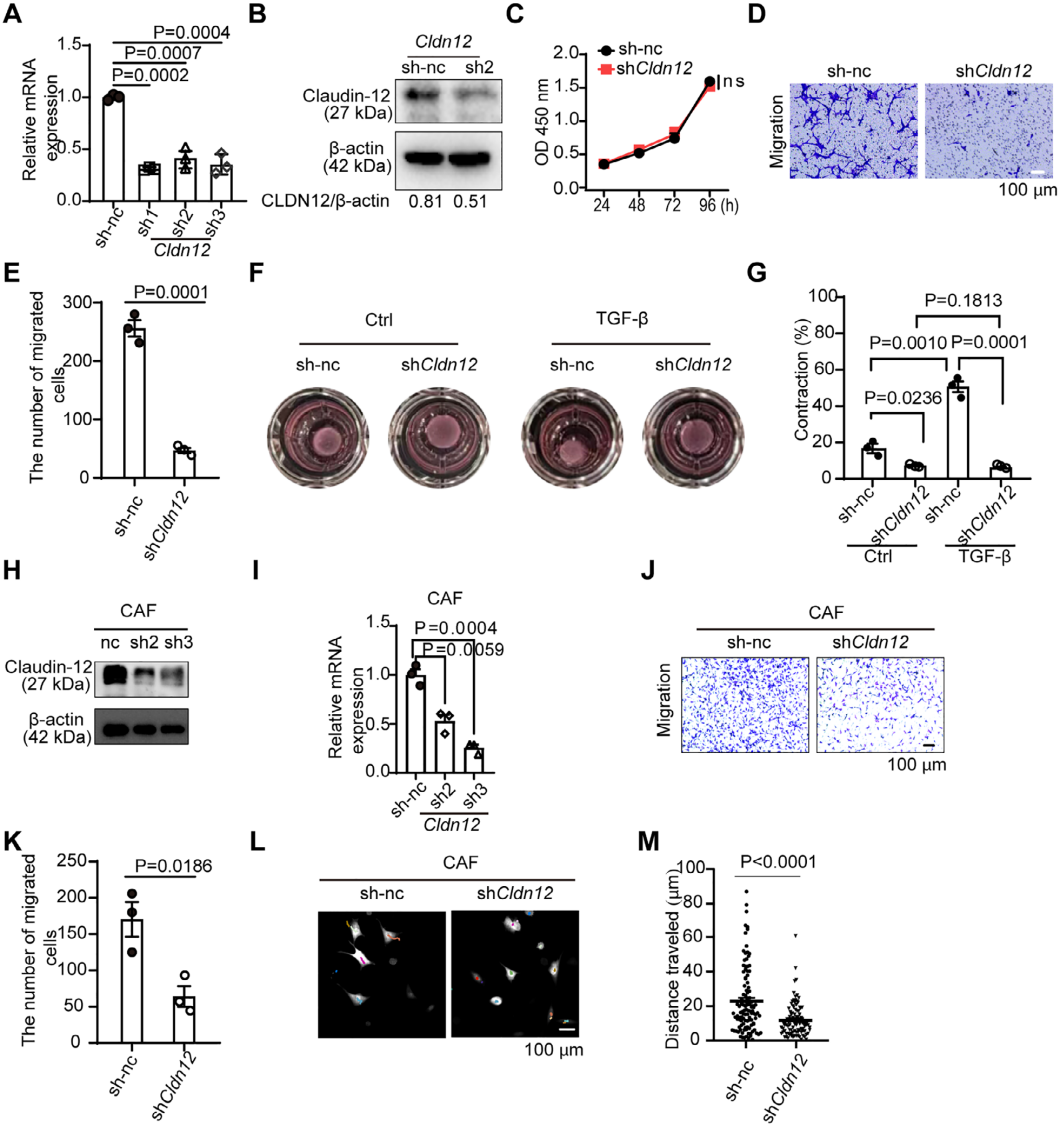
CLDN12 enhances the migration and contraction capability of fibroblasts. (A) qPCR analysis of *Cldn12* mRNA expression in iMEF cells transfected with shRNA targeting *Cldn12* (sh1, sh2, sh3) or the control shRNA (sh‐nc). *N* = 3, one‐way ANOVA. (B) Western blot analysis of CLDN12 protein expression in iMEF^sh‐nc^ and iMEF^sh^ *^Cldn12^* (sh2). (C) The proliferation of iMEF^sh‐nc^ and iMEF^sh^ *^Cldn12^* detected by CCK‐8 (*n* = 3, two‐way ANOVA). (D, E) The migration of iMEF^sh‐nc^ and iMEF^sh^ *^Cldn12^* cells analyzed by Transwell assay. For each well, 3–9 random fields were imaged and averaged, with three replicate wells per group. Scale bar: 100 µm. *N* = 3 for iMEF^sh‐nc^, *N* = 3 for iMEF^sh^ *^Cldn12^*, *t*‐test. (F, G) Representative images and quantification of gel contraction from embedded iMEF^sh‐nc^ and iMEF^sh^ *^Cldn12^* cells treated with or without TGF‐β (10 ng/mL) for 48 h. Images were taken with a digital camera and the changes in collagen gel area were calculated using ImageJ. The percentage of gel shrinkage was calculated using the formula: [(initial gel area—area after contraction) / initial area] × 100%. *N* = 3 for each group, *t*‐test. (H, I) The expression of CLDN12 protein (H) and *Cldn12* mRNA (I) in CAF^sh‐nc^ and CAF^sh^ *^Cldn12^*. *N* = 3 for each group, one‐way ANOVA. (J, K) The migration of CAF^sh‐nc^ and CAF^sh^ *^Cldn12^* cells detected by Transwell assay. *N* = 3 for each group. Scale bar: 100 µm. *t*‐test. (L) Representative time‐lapse microscopy images showing the local motion of CAF^sh‐nc^ and CAF^sh^ *^Cldn12^* cells. The motion trajectory of an individual cell was tracked by ImageJ and represented by a color line. The motion distance of individual cell was measured by ImageJ. Scale bar: 100 µm. (M) Quantification of the motion distance of CAF*‐Cldn12* ^sh‐nc^ (*n* = 111) and CAF^sh^ *^Cldn12^* (*n* = 100) cells. *t*‐test.

### 2.3 CLDN12 Promotes Physical Interactions Between Tumor Cells and CAFs

As CLDN12 was found to be expressed in CAFs, we next examined how CLDN12‐positive CAFs contribute to cancer progression. CLDN12 can form homotypic CLDN12–CLDN12 interactions to mediate cell–cell adhesion [2, 16]. The expression of CLDN12 in pancreatic cancer Pan02 cells was confirmed (Figure S1B, C). The knockdown of CLDN12 in Pan02 cells reduced the proliferation and migration of cancer cells (Figure S1D‐K), consistent with a previous study [15]. To determine whether CLDN12 mediates fibroblast‐tumor adhesion, an adhesion assay was performed (Figure 3A). GFP‐expressing iMEF^sh^ *^Cldn12^* and iMEF^sh‐nc^ fibroblasts were co‐cultured with Pan02 cells, and the proportion of adherent cells was quantified by flow cytometry. The results showed that iMEF^sh^ *^Cldn12^* cells adhered to Pan02 cells significantly less than iMEF^sh‐nc^ cells (Figures 3B, C), indicating that CLDN12 was involved in fibroblast–cancer cell adhesion. Fluorescence microscopy showed similar results (Figure 3D, E). To verify whether this effect was consistent in an alternative cancer cell line, we examined the adhesion between iMEF cells and another pancreatic cancer line, KPC‐Luc. The adhesion of iMEF^sh^ *^Cldn12^* to KPC‐Luc was also reduced compared with iMEF^sh‐nc^ (Figure S2A, B). To more closely imitate the highly complex and dynamic physiological environment in vivo, we isolated primary MEFs from *Cldn12* ^+/+^ and *Cldn12* ^−/−^ mice [16]. As expected, *Cldn12* mRNA was undetectable in *Cldn12* ^−/−^ MEFs (Figure S2C). When co‐cultured with Pan02‐GFP‐Luc cells, the adhesion of tumor cells to Cldn12^+/+^ MEFs was significantly higher than that to Cldn12^−/−^ MEFs (Figure 3F, G), confirming the requirement of CLDN12 for fibroblast‐tumor cell attachment. Next, we investigated whether CLDN12 affects the adhesion between CAFs and Pan02 cells. The adhesion of CAF^sh‐nc^ and CAF^sh^ *^Cldn12^* (sh2, sh3) cells to Pan02 was measured, and the results showed a marked reduction in adhesion following CLDN12 knockdown in CAFs (Figure S2D, E), consistent with the results obtained in MEFs. To assess direct cell–cell adhesion, fibroblasts, and tumor cells were differentially labeled, mixed in suspension, and incubated to allow physical contact. Adhesion events were quantified by flow cytometry based on the detection of CellTrace Violet^+^/PKH26^+^ double‐positive populations, which represent tumor cell–fibroblast adhesion events. The experimental workflow and gating strategy are shown in Figures 3H and S2F, respectively. Given that CLDN12 may mediate homophilic interactions between fibroblasts and tumor cells, we next performed reciprocal knockdown experiments. Silencing CLDN12 in either CAFs, Pan02 cells, or both markedly reduced the adhesion between the two cell types (Figure 3I), supporting a CLDN12–CLDN12‐dependent interaction. To further confirm this, CAF‐NC or OE (overexpression) cells were labeled with CellTrace Violet and Pan02 cells with PKH26, following the same experimental procedure as described in Figure 3H. The overexpression of CLDN12 in CAFs was confirmed by Western blotting (Figure S2G). When CLDN12 was overexpressed in CAFs, the proportion of double‐positive cells markedly increased compared with the control group, indicating that elevated CLDN12 expression strengthens homophilic CLDN12–CLDN12 interactions and promotes fibroblast‐tumor cell adhesion (Figure 3J, K). To directly assess whether CLDN12 molecules expressed by tumor cells and CAFs physically interact, Pan02 cells expressing CLDN12‐GFP were co‐cultured with CAFs expressing CLDN12‐mCherry. Following immunoprecipitation of GFP‐tagged CLDN12, CLDN12‐mCherry was specifically detected in the immunoprecipitated complex, whereas no signal was observed in the IgG control, indicating a physical association between tumor cell‐derived and CAF‐derived CLDN12 molecules (Figure 3L). These results provide biochemical evidence supporting homophilic CLDN12–CLDN12 interactions between tumor cells and fibroblasts. We next examined whether CLDN12‐mediated interactions occur within the tumor microenvironment. Immunofluorescence staining of mouse pancreatic tumors further supported this observation. At sites where blue pan‐CK^+^ tumor cells and red α‐SMA^+^ CAFs were in direct contact, strong green CLDN12 signals were detected, suggesting that CLDN12 participates in cancer‐CAF physical interactions within the tumor microenvironment (Figure 3M). Finally, to directly visualize homophilic CLDN12 interactions, Pan02 cells overexpressing CLDN12‐GFP and CAFs overexpressing CLDN12‐mCherry were co‐cultured in vitro, with GFP and mCherry serving as reporters of CLDN12 localization on tumor cells and CAFs, respectively. Confocal microscopy revealed prominent co‐localization of GFP and mCherry signals at cell–cell contact sites, appearing as merged yellow signals at the tumor‐CAF interface, providing direct visual evidence of CLDN12–CLDN12 homotypic interactions (Figure 3N). Collectively, these results demonstrate that CLDN12 promotes the adhesion of fibroblasts to pancreatic cancer cells through homophilic CLDN12–CLDN12 interactions.

**FIGURE 3:**
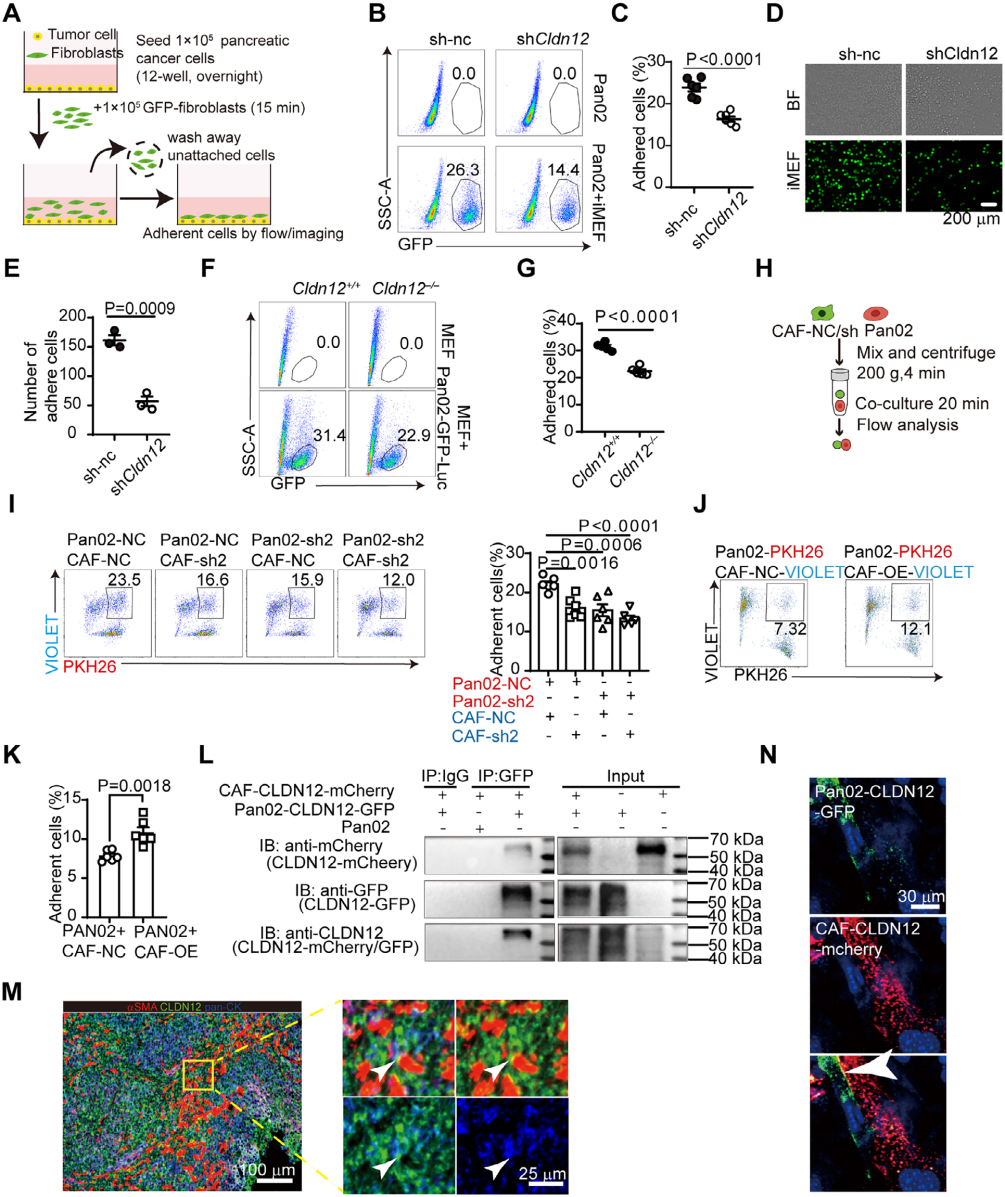
CLDN12 mediates the adhesion of fibroblasts to pancreatic cancer cells. (A) Schematic illustration of the plate‐based cell–cell adhesion assay used to assess direct interactions between fibroblasts and pancreatic cancer cells. (B, C) The adhesion of iMEF cells to Pan02 cells detected by flow cytometry. iMEF^sh‐nc^ and iMEF^sh^ *^Cldn12^* cells were added onto Pan02 cell monolayer for 15 min. Unattached cells were washed away. *N* = 6 for each group, *t*‐test. (D, E) The adhesion of iMEF^sh‐nc^ and iMEF^sh^ *^Cldn12^* cells to Pan02 cells detected by fluorescence microscopy. The adhered fibroblasts in each high‐power field (HPF) were counted by ImageJ. *N* = 3 for each group, scale bar: 200 µm. (F, G) The adhesion of Pan02‐GFP‐Luc cells to *Cldn12* ^+/+^ and *Cldn12* ^−/−^ MEFs detected by flow cytometry. *N* = 5 for each group, *t*‐test. (H) Schematic illustration of the flow cytometry‐based CAF‐Pan02 adhesion assay. CAFs and Pan02 cells were differentially labeled with CellTrace Violet and PKH26, respectively, co‐incubated, and analyzed by flow cytometry. CellTrace Violet^+^/PKH26^+^ double‐positive populations were used as a readout for tumor cell–fibroblast adhesion events. (I) Representative flow cytometric plots and quantitative analysis of CAF‐Pan02 adhesion after CLDN12 knockdown. Four co‐culture groups were tested: CAF^sh‐nc^ + Pan02^sh‐nc^, CAF ^sh^ *^Cldn12^* + Pan02^sh‐nc^, CAF^sh‐nc^ + Pan02^sh^ *^Cldn12^*, and CAF^sh^ *^Cldn12^* + Pan02^sh^ *^Cldn12^*. *N* = 5 for each group, one‐way ANOVA. (J, K) Representative flow cytometry plots and quantitative analysis of adhesion between CAF‐NC/OE CLDN12 cells and Pan02 cells. *N* = 6 for each group, *t*‐test. (L) Co‐immunoprecipitation analysis of CLDN12–CLDN12 interaction between pancreatic cancer cells and CAFs. Pan02 cells expressing CLDN12‐GFP were co‐cultured with CAFs expressing CLDN12‐mCherry, followed by immunoprecipitation with anti‐GFP antibody. The presence of CLDN12‐mCherry in the immunoprecipitated complex was detected by immunoblotting. (M) Immunofluorescence staining of pancreatic tissues from the orthotopic pancreatic tumor model. Pan‐cytokeratin (pan‐CK, blue) marks epithelial tumor cells, Red: α‐SMA^+^ fibroblasts (CAFs); green: CLDN12. Scale bar: 100 µm (low magnification) and 25 µm (high magnification). (N) Representative fluorescence images showing the co‐localization of CLDN12‐GFP (tumor cells) and CLDN12‐mCherry (fibroblasts) at cell–cell junctions. Three to nine fields were captured per group, and the experiment was repeated three times independently. Scale bar: 30 µm.

### 2.4 CLDN12‐Expressing CAFs Promote the Invasion of Pancreatic Cancer Cells

Previous studies have shown that direct adhesion between cancer cells and fibroblasts can promote fibroblast‐guided cancer cell invasion [22, 24]. Based on this framework, we next examined whether CLDN12‐mediated cancer cell–fibroblast adhesion influences the invasive behavior of pancreatic cancer cells. First, the migration of Pan02‐mCherry cells co‐cultured with iMEF^sh‐nc^ and iMEF^sh^ *^Cldn12^* cells in the upper chamber of Transwell was examined (Figure 4A). After 24 h, the migrated Pan02‐mCherry cells co‐cultured with iMEF^sh‐nc^ cells largely increased compared to Pan02‐mCherry alone. In contrast, the migrated Pan02‐mCherry cells co‐cultured with iMEF^sh^ *^Cldn12^* cells decreased compared to those co‐cultured with iMEF^sh‐nc^ cells (Figure 4B, C), suggesting that the expression of CLDN12 in fibroblasts contributes to enhanced tumor‐cell migration. To examine cancer cell invasion, the mixture of Pan02‐mCherry cells and iMEF cells was co‐cultured to form a monolayer, and then a wound was created by a scratch following a cover of Matrigel. After 24 h, invasion of both fibroblasts and cancer cells into the wound area was quantified. Pan02 tumor cells co‐cultured with iMEF^sh‐nc^ displayed greater invasion than those with iMEF^sh^ *^Cldn12^* cells. A similar reduction in invasion was observed for iMEF^sh^ *^Cldn12^* fibroblasts themselves (Figure 4D, E), suggesting that loss of CLDN12 attenuates both fibroblast and tumor invasiveness. We further examined invasion using a 3D spheroid model. Pan02‐mCherry cells were mixed with CAF^sh‐nc^ or CAF^sh^ *^Cldn12^* cells in methylcellulose‐containing droplets to form spheroids, which were then embedded in Matrigel. After 24 h, the invasion of matrix by fibroblasts and cancer cells was evaluated. Pan02‐mCherry and CAF^sh^ *^Cldn12^* spheroids displayed smaller matrix‐invading areas than those formed with CAF^sh‐nc^ cells (Figure 4F, G). In Figure 4F, more CAF^sh‐nc^ fibroblasts (green) migrated beyond the spheroid boundary, leaving a larger proportion of tumor cells (red) inside the spheroid core, consistent with the notion that fibroblast CLDN12 enhances fibroblast invasion, which in turn facilitates tumor‐cell dissemination. Correspondingly, tumor outgrowth area from spheroids was smaller for Pan02‐mCherry co‐cultured with CAF^sh^ *^Cldn12^* cells than for those co‐cultured with CAF^sh‐nc^ cells (Figure 4F, H). To directly observe fibroblast‐guided cancer cell invasion, CAF^sh‐nc^ or CAF^sh^ *^Cldn12^* cells were co‐cultured with Pan02‐mCherry cells in Matrigel, and then time‐lapse microscopy imaging was performed to track cell movement for 3 h. Among cancer cells physically associated with fibroblasts, both fibroblast and tumor‐cell motility were substantially reduced when CLDN12 was depleted (Figure 4I). Collectively, these results suggest that CLDN12 expression in fibroblasts enhances their ability to guide and promote the invasion of pancreatic cancer cells.

**FIGURE 4:**
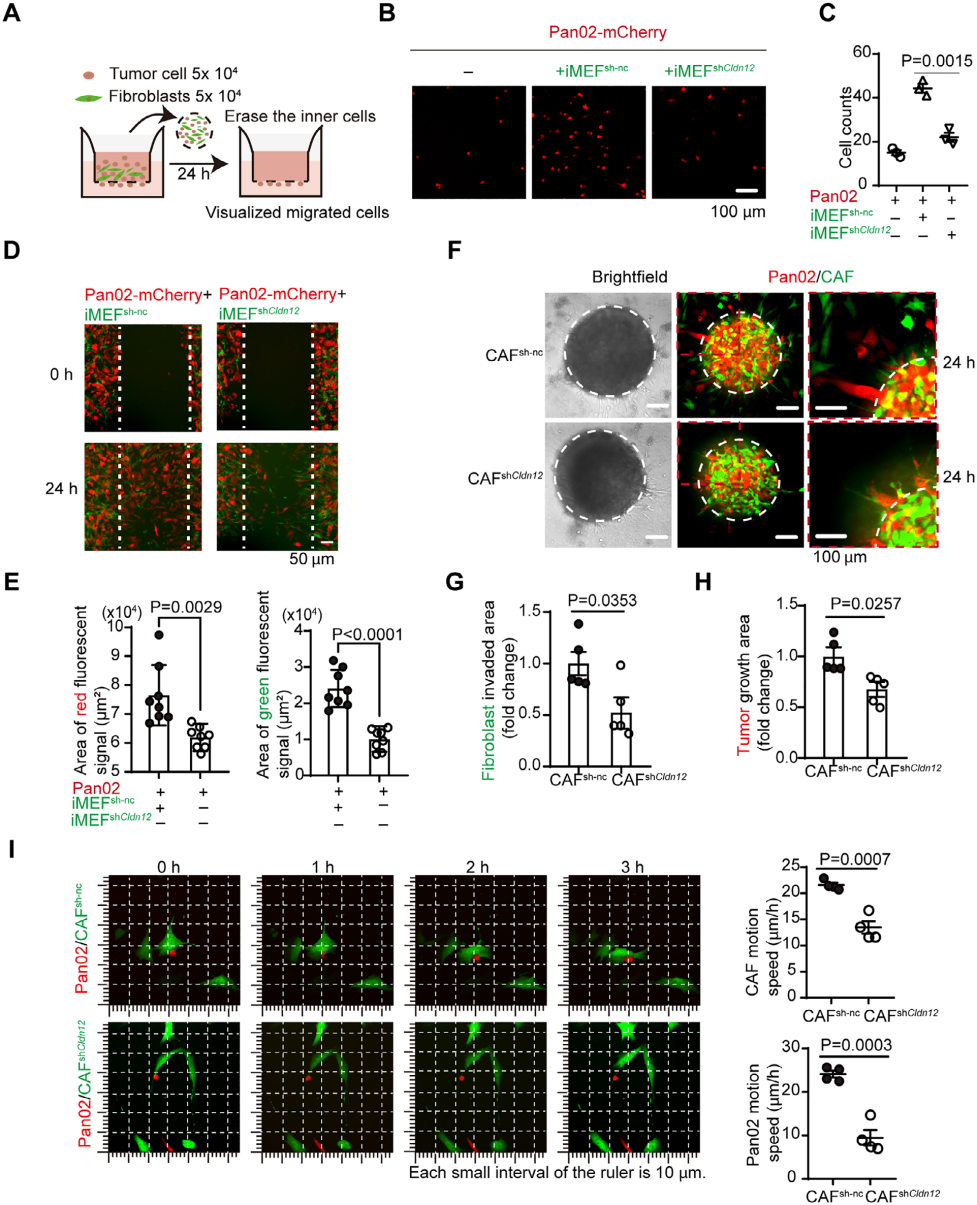
CLDN12‐expressing fibroblasts enhance the migration and invasion of pancreatic cancer cells. (A) Schematic of the Transwell migration assay in which Pan02‐mCherry cells were co‐cultured with fibroblasts. (B, C) Representative images (B) and quantitative analysis (C) of Pan02‐mCherry cell migration after co‐culture with iMEFsh‐nc or iMEFshCldn12 cells. The number of migrated tumor cells per high‐power field (HPF) was quantified using ImageJ. *N* = 3 for each group, one‐way ANOVA. Scale bar: 100 µm. (D, E) 2D invasion assay was performed with Pan02‐mCherry cells co‐cultured with iMEF^sh‐nc^ or iMEF^sh^ *^Cldn12^* cells in Matrigel. The area of Pan02‐mCherry and iMEF cells invading into the wound area at 24 h was quantified. Scale bar, 50 µm. *N* = 8 for each group, *t*‐test. (F–H) The outgrowth of Pan02‐mCherry cells co‐cultured with CAF^sh‐nc^ or CAF^sh^ *^Cldn12^* cells from 3D spheroids in Matrigel matrix. Pictures were taken after 24 h. Fibroblast invasion area per spheroid, and tumor outgrowth area were normalized to total spheroid area and expressed as fold change relative to the control. *N* = 5 for each group, *t*‐test, scale bar: 100 µm. (I) Time‐lapse microscopy imaging of Pan02 tumor cells (red) co‐cultured with CAF^sh‐nc^ or CAF^sh^ *^Cldn12^* cells (green). Cell motion distance was tracked by ImageJ and the motion speed was calculated. *N* = 4 for each group, *t*‐test. Each small interval of the ruler is 10 µm.

### 2.5 TGF‐β Upregulates CLDN12 in CAFs

Given that CLDN12 mediates cancer cell–fibroblast adhesion, we next sought to determine whether tumor‐derived signals regulate CLDN12 expression in fibroblasts. iMEF cells were treated with pancreatic cancer cell‐derived conditioned medium (Pan02‐CM), which markedly increased *Cldn12* mRNA expression (Figure 5A). To identify the soluble factors responsible for this induction, we screened a panel of candidate cytokines and found that TGF‐β robustly upregulated Cldn12 in iMEFs (Figure 5B, C). Additional analyses showed that TNF‐α, IFN‐γ, and S100A4 had no effect, while GM‐CSF significantly upregulated CLDN12 expression in iMEFs, although to a lesser magnitude compared with TGF‐β (Figure S3A‐D). Considering the established role of TGF‐β as a central regulator of fibroblast activation [30], subsequent experiments focused on TGF‐β‐mediated regulation of CLDN12. Pan02 and KPC cancer cells expressed and secreted TGF‐β, as detected in both cell lysates and culture supernatants collected at 24 h and 48 h (Figure 5D,E). Importantly, Pan02‐CM activated TGF‐β signaling in iMEFs, as evidenced by increased SMAD2 phosphorylation (Figure 5F). To determine whether TGF‐β directly regulates CLDN12 expression, CAFs were treated with recombinant TGF‐β in the presence or absence of the TGF‐β receptor inhibitor SD208. SD208 markedly attenuated TGF‐β‐induced *Cldn12* upregulation (Figure 5G), indicating that CLDN12 induction is mediated through TGF‐β receptor signaling. Consistently, recombinant TGF‐β increased CLDN12 protein expression (Figure S3E). In addition, SD208 treatment suppressed Pan02‐CM‐induced CLDN12 protein expression in iMEFs (Figure 5H), supporting that tumor cell‐derived soluble factors induce CLDN12 expression through TGF‐β‐dependent signaling. Functionally, Pan02‐CM enhanced the adhesion of Pan02‐GFP‐Luc cells to iMEFs, whereas this effect was significantly reduced by SD208 treatment (Figure 5I, J). Similarly, recombinant TGF‐β promoted cancer cell–fibroblast adhesion, and this effect was abolished by SD208 treatment (Figure S3F, G). Together, these results indicate that TGF‐β receptor signaling is a major mediator of Pan02‐CM‐induced CLDN12 expression and the associated enhancement of cancer cell–fibroblast adhesion.

**FIGURE 5:**
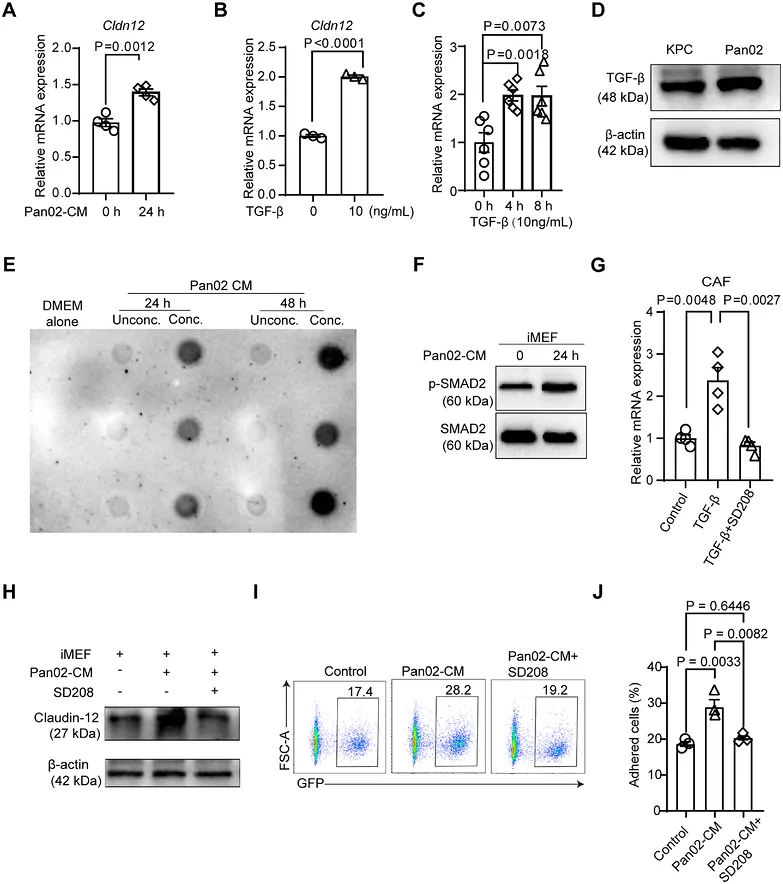
TGF‐β signaling mediates tumor‐derived induction of CLDN12 in fibroblasts. (A) The expression of *Cldn12* mRNA in iMEF cells treated with Pan02‐CM for 24 h. *N* = 4 for each group, *t*‐test. (B) The expression of *Cldn12* mRNA in iMEF cells treated with TGF‐β (10 ng/mL) for 24 h. *N* = 3 for each group, *t*‐test. (C) The expression of *Cldn12* mRNA in iMEF cells treated with TGF‐β (10 ng/mL) for 4 and 8 h. *N* = 6 for each group, one‐way ANOVA. (D) The expression of TGF‐β in pancreatic cancer cell lines (KPC, Pan02) detected by Western blotting. (E) Dot‐blot analysis of secreted TGF‐β levels in unconcentrated (Unconc.) and concentrated (Conc.) media collected from Pan02 cells at 24 h and 48 h. (F) The phosphorylation level of SMAD2 in iMEF cells treated with Pan02‐CM for 24 h was detected by Western blotting. (G) CAFs were treated with TGF‐β (10 ng/mL) with or without SD208 (2 µM) for 24 h, and *Cldn12* expression was analyzed by qPCR. *N* = 4 for each group, one‐way ANOVA. (H) iMEF cells were treated with Pan02‐CM with or without SD208 for 24 h, and CLDN12 protein expression was analyzed by Western blotting. (I, J) Pan02‐GFP‐Luc cells and iMEF cells were co‐cultured in the presence of Pan02‐CM with or without SD208(2 µM, 24 h) treatment, and cancer cell–fibroblast adhesion was analyzed by flow cytometry‐based adhesion assay (I). Quantification of adhesion events is shown in J. *N* = 3 for each group, one‐way ANOVA.

### 2.6 CLDN12‐Expressing CAFs Promote Pancreatic Cancer Metastasis In Vivo

To determine whether CLDN12‐expressing fibroblasts influence tumor progression in vivo, we first established a subcutaneous xenograft model in wild‐type C57BL/6 mice. Pan02‐GFP‐Luc cells were subcutaneously (s.c.) co‐injected with iMEF^sh‐nc^ or iMEF^sh^ *^Cldn12^* fibroblasts (Figure S4A). Notably, *Cldn12* knockdown was performed in fibroblasts before co‐injection, thereby specifically reducing fibroblast‐derived CLDN12 without altering CLDN12 expression in tumor cells. As the subcutaneous site might provide a relatively interaction‐poor environment for pancreatic cancer, no significant difference in tumor growth was observed between the groups (Figure S4B, C). Given our in vitro findings that CLDN12 enhances fibroblast‐tumor cell adhesion, we next evaluated tumor progression in an orthotopic pancreatic implantation model (Figure 6A). In this model, mice receiving iMEF^sh^ *^Cldn12^* fibroblasts exhibited reduced primary tumor burden, as assessed by ex vivo bioluminescence imaging (Figure S4D, E), and showed markedly reduced liver and diaphragm metastases (Figures 6B‐D and S4F) and a decreased incidence of colon metastasis (Figure S4G, H) compared with control fibroblast‐co‐implanted mice. The finding that fibroblast‐specific CLDN12 depletion weakened tumor–fibroblast interactions in vitro and concurrently reduced metastatic dissemination in vivo, together with the clinical reality that metastatic spread is the major cause of mortality in pancreatic cancer, prompted us to further investigate the specific contribution of CLDN12 to metastatic colonization. Next, cancer cells and fibroblasts were mixed and co‐injected into the spleen to establish a liver metastasis model of pancreatic cancer (Figure 6E). Bioluminescence imaging revealed that the liver metastasis of Pan02‐GFP‐Luc cells mixed with iMEF^sh^ *^Cldn12^* cells was apparently decreased compared with that in the iMEF^sh‐nc^ cells (Figure 6F‐H). H&E staining confirmed significantly fewer metastatic foci in iMEF^sh^ *^Cldn12^* group (Figure 6I, J). Similarly, co‐injection of KPC‐Luc cells with CAF^sh^ *^Cldn12^* cells led to reduced liver metastasis compared to the CAF^sh‐nc^ group (Figure 6K, L). Collectively, these results indicate that fibroblast‐derived CLDN12 markedly promotes pancreatic cancer metastasis in vivo.

**FIGURE 6:**
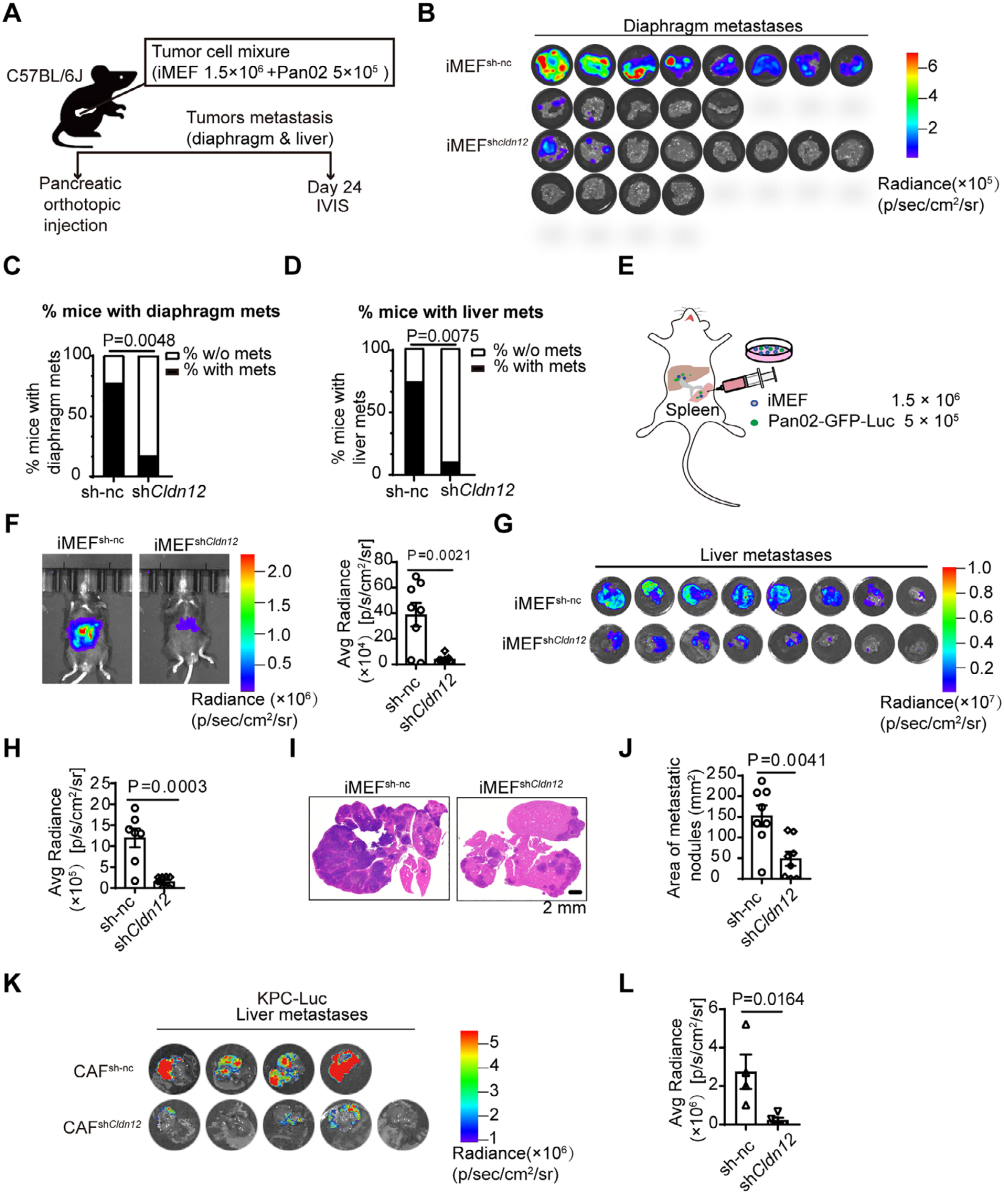
CLDN12‐expressing fibroblasts promote metastasis of pancreatic cancer in vivo. (A) Schematic illustration of the orthotopic pancreatic cancer model. Pan02‐GFP‐Luc pancreatic cancer cells were mixed with iMEF^sh‐nc^ or iMEF^sh^ *^Cldn12^* fibroblasts and orthotopically injected into the pancreas of wild‐type C57BL/6 mice. Tumor growth and metastatic dissemination were monitored by bioluminescence imaging, and tissues were harvested 24 days after implantation. (B) Representative ex vivo bioluminescence images showing metastatic lesions in the diaphragm. (C, D) Quantification of the incidence of liver and diaphragm metastasis. The iMEF^sh‐nc^ group included 13 mice, and the iMEF^sh^ *^Cldn12^* group included 12 mice. Statistical analysis was performed using Fisher's exact test. (E) Schematic illustration of liver metastasis model of pancreatic cancer. Pan02‐GFP‐Luc cells mixed with iMEF^sh‐nc^ and iMEF^sh^ *^Cldn12^* cells in Matrigel were injected into the spleen of naive C57BL/6 mice. (F) Representative images showing in vivo luminescence of liver metastasis of Pan02‐GFP‐Luc co‐injected with iMEF^sh‐nc^ and iMEF^sh^ *^Cldn12^* cells measured at 2 weeks after tumor injection. Quantification of the luminescence intensity of liver metastasis in vivo. *n* = 8 per group, *t*‐test. (G, H) Liver metastasis measured by IVIS system and luminescence intensity was quantified. *n* = 8 per group, *t*‐test. (I) Representative H&E staining of liver metastasis. Scale bar: 2 mm. (J) Quantification of tumor metastasis area in liver. *n* = 8 mice per group, *t*‐test. (K, L) Representative IVIS images and quantification of metastatic liver signals from KPC‐Luc co‐injection with CAF^sh‐nc^ and CAF^sh^ *^Cldn12^* cells. *n* = 4 for CAF^sh‐nc^; *n* = 5 for CAF^sh^ *^Cldn12^*; *t‐*test.

### 2.7 Targeting CLDN12 by C12‐pm Reduces Liver Metastasis of Pancreatic Cancer

As the extracellular loop 2 (ECL2) of CLDNs often mediates the homotypic interaction between adjacent cells [31], we designed a peptide mimic corresponding to ECL2 of CLDN12 (C12‐pm: YNSHLNRKFEP) and a control peptide (Con‐p: LYQY) [32]. Cancer cells or fibroblasts were pretreated with C12‐pm (400 µM) to compete with endogenous CLDN12 for 24 h, and then the two types of cells were co‐cultured to examine their adhesion. The pretreatment of either Pan02 cells or fibroblasts with C12‐pm effectively inhibited the adhesion between iMEFs and cancer cells (Figures 7A, B and S5A, B). To further examine whether C12‐pm‐mediated inhibition of adhesion requires CLDN12, Pan02^sh2^ and CAF^sh2^ cells, with or without CLDN12 knockdown, were co‐cultured in various combinations. C12‐pm treatment significantly reduced cell adhesion in control groups, whereas this effect was lost when CLDN12 was knocked down in either Pan02 or CAFs (Figure 7C, D). Because TGF‐β could upregulate CLDN12 in fibroblasts, TGF‐β‐stimulated CAFs were pretreated with C12‐pm, and the adhesion of Pan02‐GFP‐Luc cells to CAFs was examined. C12‐pm largely prevented the adhesion of Pan02‐GFP‐Luc cells to TGF‐β‐stimulated CAFs (Figures 7E and S5C). Finally, we evaluated the therapeutic potential of C12‐pm in vivo. Mice were co‐injected with Pan02‐GFP‐Luc and CAF cells into the spleen to establish a liver metastasis model, followed by tail‐vein injection of C12‐pm or Con‐p every 2 days for six doses (Figure 7F). Bioluminescence imaging revealed that C12‐pm treatment markedly reduced liver metastasis compared with controls (Figure 7G, H). H&E staining further confirmed a significant decrease in metastatic foci in C12‐pm–treated mice (Figure 7I, J). Together, these findings demonstrate that C12‐pm disrupts CLDN12‐dependent adhesion between fibroblasts and pancreatic cancer cells, thereby suppressing fibroblast‐driven liver metastasis.

**FIGURE 7:**
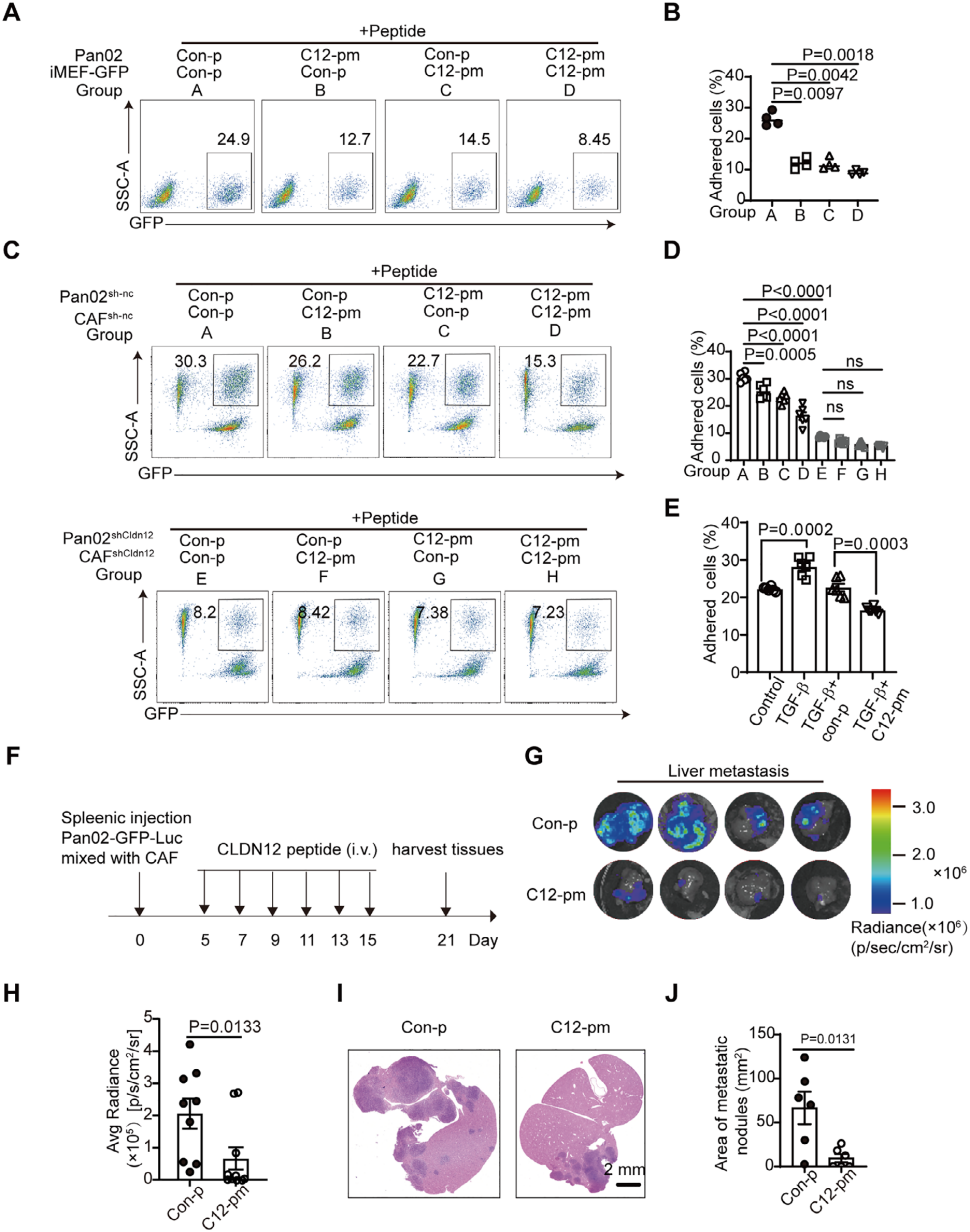
CLDN12 peptide mimics (C12‐pm) inhibit fibroblast‐mediated adhesion and liver metastasis of pancreatic cancer. (A, B) Flow cytometric analysis of iMEF adhesion to Pan02 cells after pretreatment with control peptide (Con‐p) or CLDN12 peptide mimics (C12‐pm, 400 µM) for 24 h. *N* = 4 per group, one‐way ANOVA. (C, D) Violet‐labeled CAFs and PKH26‐labeled Pan02 cells were mixed all possible combinations of Con‐P/C12‐pm‐treated pairs: (A‐D) CAF^sh‐nc^/Pan02^sh‐nc^ and (E‐H) CAF^sh^ *^Cldn12^*/Pan02^sh^ *^Cldn12^*. After gentle centrifugation (200 g, 4 min) and co‐incubation at 37°C for 20 min, double‐positive cells were measured by flow cytometry. *N* = 6 per group, one‐way ANOVA. (E) Adhesion of Pan02‐GFP‐Luc cells to TGF‐β–stimulated (10 ng/mL) CAFs pretreated with Con‐p or C12‐pm (400 µM) for 24 h. *N* = 6*, t*‐test. (F) Schematic illustration of in vivo peptide treatment. (G, H) Representative bioluminescence images and quantification of liver metastasis in mice co‐injected with Pan02‐GFP‐Luc and CAFs, followed by tail‐vein injection of Con‐p or C12‐pm every 2 days for six times. Liver metastasis was assessed by bioluminescence imaging on day 21. *n* = 9 for Con‐p; *n* = 10 for C12‐pm; *t*‐test. (I, J) Representative H&E‐stained images of liver sections from Con‐p and C12‐pm‐treated mice showing metastatic foci. Scale bar, 2 mm. Quantification of metastatic area revealed a significant reduction in C12‐pm‐treated group. *n* = 6 per group, *t*‐test.

To further assess the clinical relevance of CLDN12 in human PDAC, we analyzed public human PDAC datasets, including bulk and single‐cell transcriptomic data. In GSE71729, CLDN12 expression was significantly higher in metastatic PDAC than in primary PDAC and normal pancreas (Kruskal–Wallis *p* = 3.84e‐12; all pairwise BH‐adjusted *p* < 0.001) (Figure S6A). Single‐cell RNA‐seq analysis of human PDAC (GSE154778) showed that CLDN12 was expressed in epithelial tumor‐like cells and CAFs (Figure S6B, C). These results support an association between CLDN12 and metastatic PDAC and confirm its expression in human CAFs.

## 3 Discussion

Distal metastasis of pancreatic cancer is prevalent and the mechanisms remain not fully understood [33]. Tight junction proteins including CLDNs are often expressed on epithelial cells to maintain tissue integrity and are usually dysregulated in pancreatic cancer [1, 4]. In human pancreatic cancer cells, CLDN12 plays a tumorigenic role and promotes the malignant phenotype of pancreatic cancer cells [15]. In this study, we found that CLDN12 is expressed in CAFs, which are usually non‐tight junction forming cells. CLDN12 expressed in fibroblasts mediates the adhesion between CAFs and pancreatic cancer cells, promoting the invasion and metastasis of pancreatic cancer. Our findings extend the current understanding of how CLDN12 functions not only in tumors but also in CAFs within the microenvironment, contributing to tumor metastasis of pancreatic cancer. Importantly, our results demonstrate that targeting CLDN12 with C12‐pm corresponding to ECL2 effectively impedes the metastasis of pancreatic cancer, suggesting a promising strategy for tumor treatment.

Our results reveal that CLDN12 is an attractive target for the treatment of pancreatic cancer. CLDNs expressed in cancer cells and stromal cells have emerged as novel targets for tumor therapy. For example, CLDN18.2‐targeted therapies are under development for the treatment of gastric, pancreatic, and esophageal cancers [5, 34]. The expression of CLDN18 on cancer cells has been reported to promote T lymphocyte infiltration and antitumor immunity in pancreatic cancer [34]. It has been reported that lung metastasis of breast cancer is facilitated by angiopoietin 2‐mediated suppression of lung‐specific endothelial CLDN5, while kidney metastasis is prevented by non‐Ang2‐responsive CLDN2 and CLDN10 [6]. Suppression of Claudins 2 and 10 was sufficient to facilitate kidney metastasis of breast cancer. CLDN12 has been found to promote the proliferation and migration of pancreatic cancer and osteosarcoma cells [15, 35]. Reduced expression of CLDN12 in cervical cancer predicts poor prognosis of patients with cervical cancer [36]. We previously reported that CLDN12 expressed on MDSCs and vascular endothelial cells promoted the accumulation of MDSCs at the tumor site through homotypic interaction [16]. In this study, we report that CLDN12 expressed on CAFs promotes the invasion of cancer cells, thereby facilitating distal metastasis of pancreatic cancer. Targeting CLDN12 blocked liver metastasis of pancreatic cancer. Therefore, CLDN12 represents a potentially promising therapeutic target for pancreatic cancer. However, several challenges remain for the clinical translation of CLDN12‐targeting strategies. CAFs are highly heterogeneous, and CLDN12‐expressing fibroblasts may represent only a subset of tumor‐promoting CAF populations. Further studies are needed to determine the expression pattern and physiological functions of CLDN12 in normal tissues and to evaluate the safety and clinical feasibility of targeting CLDN12‐mediated stromal interactions. In addition, public human PDAC datasets support an association between CLDN12 expression and metastatic PDAC and confirm CLDN12 expression in human CAFs, but do not currently support CLDN12 as a prognostic biomarker.

Our study demonstrates that CLDN12‐expressing CAFs physically interact with cancer cells and promote distal metastasis of pancreatic cancer by promoting the invasion of cancer cells. Our findings and those of others suggest that CAFs play a key role in the progression, invasion, and metastasis of cancers [20, 26, 37]. Although many studies demonstrate that CAFs communicate with cancer cells by secreting a variety of soluble factors or exosomes [20, 38, 39], pioneering studies also showed that CAFs make direct cell–cell contact with cancer cells, remodeling their behavior, such as metastasis or chemotherapy resistance [22, 24, 26]. CLDN12 is generally expressed in tumor cells, and its expression in fibroblasts suggests frequent interaction between fibroblasts and tumor cells. Consistent with this, CLDN12 may play an important role in fibroblasts. CAFs could exert a physical force on cancer cells that enables their collective invasion through heterotypic E‐cadherin/N‐cadherin adhesion [22]. In this study, our results demonstrate that CLDN12 mediates the direct adhesion between fibroblasts and pancreatic cancer cells, promoting liver metastasis of pancreatic cancer through homotypic CLDN12 interaction. CLDN12 undergoes four transmembrane transitions, forming two extracellular loops (ECL1 and ECL2) and an intracellular loop, with N‐terminal and C‐terminal tail located in the cytoplasm, and it does not have a PDZ domain [8, 9]. The larger ECL1 loop is mainly involved in the selectivity of intercellular ions, while the smaller ECL2 loop can interact with ECL2 of CLDN molecules expressed on adjacent cells [16]. In this study, the results suggest that the knockdown of CLDN12 in fibroblasts or cancer cells reduced their adhesion. Synthetic competing peptides corresponding to ECL2 could impair CLDN12‐mediated CAF–cancer cell adhesion, suggesting a homotypic CLDN12 interaction between them. Since CLDN12‐mediated adhesion is driven by its homophilic interaction, this mechanism reflects the intrinsic property of CLDN12 rather than a CAF‐specific feature, supporting the use of iMEFs as a fibroblast model for mechanistic studies. Nevertheless, iMEFs cannot fully recapitulate the heterogeneous properties of tumor‐derived CAFs, including differences in gene expression, secretory phenotypes, and extracellular matrix remodeling capacity [21, 38]. Although most relevant functional experiments in this study were performed using primary CAFs, further validation of the findings obtained with iMEFs using additional primary CAF populations is warranted. This mechanism offers a novel approach for the regulation of cancer cells by CAFs through direct contact. In our orthotopic pancreatic tumor model, knockdown of CLDN12 in fibroblasts reduced both primary tumor size and metastatic burden. Although the mechanisms underlying the decrease in primary tumor were not further explored, this finding suggests that fibroblast‐derived CLDN12 may contribute to tumor expansion in addition to promoting invasion and metastasis. This effect could arise from enhanced tumor–fibroblast interactions or other CLDN12‐dependent stromal mechanisms. Given that metastatic dissemination is the major determinant of mortality in pancreatic cancer, our study focused on clarifying the role of fibroblast‐expressed CLDN12 in metastasis. Across three independent in vivo models‐an orthotopic pancreatic cancer model and two splenic injection liver metastasis models using Pan02 and KPC tumor cells‐we consistently observed that fibroblast‐expressed CLDN12 facilitates liver metastatic colonization.

The expression of CLDN12 in CAFs is tightly regulated by cancer‐derived factors. Our results suggest that TGF‐β produced by cancer cells upregulates the expression of CLDN12 in CAFs by activating SMAD2. The TGF‐β signaling pathway plays an important role in the induction of CAFs [40]. Studies have shown that TGF‐β upregulates CLDN4 in brain glioma [41]. In this study, the results showed that pancreatic cancer cells could produce TGF‐β, which promoted the expression of CLDN12. The inhibitor SD208 attenuated the upregulation of CLDN12 by TGF‐β. Considering that CLDN12‐expressing CAFs could promote liver metastasis of pancreatic cancer, this TGF‐β‐CLDN12 axis represents a pro‐metastatic reprogramming of CAFs by cancer cells. Other tumor‐derived factors might also contribute to the modulation of CLDN12 in CAFs, which requires further study.

In conclusion, our findings reveal a hitherto unreported mechanism that influences pancreatic cancer metastasis, in which homotypic interactions between CLDN12 molecules expressed by CAFs and pancreatic cancer cells promote intercellular adhesion and collective invasion. Targeting ECL2 of CLDN12 with C12‐pm effectively blocked the promoting effect of CLDN12 on liver metastasis of pancreatic cancer. These intriguing findings suggest a promising strategy for pancreatic cancer treatment by disrupting the homotypic interaction of CLDN12 to prevent metastasis.

## 4 Materials and Methods

### 4.1 Cell Lines and Cell Culture

Immortalized mouse embryonic fibroblasts (iMEFs) were gifted from Professor Xin Lin (Tsinghua University). The KPC‐Luc cell line was derived from spontaneous pancreatic carcinoma in LSL‐Kras^G12D/+^, LSL‐Trp53^R172H/+^, Pdx1‐Cre (KPC) mice and was provided by Dr. Xiaomeng Liu (Fudan University). Pan02, Pan02‐GFP‐Luc, KPC‐Luc, SVEC4‐10, and all fibroblasts used were cultured in DMEM medium (C11995500BT, Gibco, China). MSC2 (an MDSC cell line) and RAW264.7 cells were maintained in RPMI‐1640 medium. All media were supplemented with 100 U/mL penicillin‐streptomycin (SV30010, HyClone, Shanghai, China) and 10% fetal bovine serum (FBS) (ST30‐33‐2, PAN‐seratech, Germany) at 37°C and 5% CO_2_. Primary mouse embryonic fibroblasts (MEFs) were isolated from C57BL/6 mice as previously described [24].

### 4.2 Cancer Cell–Fibroblast Adhesion Assay

Cancer cell–fibroblast adhesion was evaluated using plate‐based and flow cytometry‐based adhesion assays. For the plate‐based assay, GFP‐labeled fibroblasts were added to pancreatic cancer cell monolayers, followed by brief co‐incubation. After removal of non‐adherent cells, adherent fibroblasts were quantified by fluorescence microscopy. For the flow cytometry‐based assay, fibroblasts and cancer cells were differentially labeled, mixed, and co‐incubated, and cell–cell adhesion was quantified based on the percentage of double‐positive events representing fibroblast–cancer cell conjugates. Detailed experimental procedures are provided in the Supplementary Information.

### 4.3 Scratch‐Invasion Assay

The invasion ability of tumor cells in the presence of fibroblasts was assessed using a Matrigel‐based scratch assay. GFP‐labeled fibroblasts and mCherry‐labeled tumor cells were co‐cultured at a 2:1 ratio, followed by scratch formation and Matrigel overlay. Images were acquired at 0 and 24 h, and wound closure and invasion distance were quantified using ImageJ.

### 4.4 Spheroid 3D Invasion Assay

Tumor cell–fibroblast co‐spheroids were generated using the hanging‐drop method as previously described [42], with minor modifications, and embedded in Matrigel. Spheroid invasion was monitored by confocal microscopy and quantified using ImageJ.

### 4.5 Transwell Migration Assay

Cell migration was assessed using 8‐µm pore Transwell inserts. For co‐culture migration assays, GFP‐labeled fibroblasts and mCherry‐labeled pancreatic cancer cells were co‐seeded in the upper chamber, whereas Pan02^sh‐nc^ and Pan02^sh^ *^Cldn12^* cells were used for single‐cell migration assays. Migrated cells were identified by fluorescence imaging or crystal violet staining, as appropriate, and quantified using ImageJ.

### 4.6 Cellular Immunofluorescence

Cells were fixed, permeabilized, and blocked before incubation with primary and appropriate fluorescent secondary antibodies. Nuclei were counterstained with DAPI, and fluorescence signals were examined by confocal microscopy. For visualization of CLDN12‐mediated cell–cell interactions, fibroblasts expressing CLDN12‐mCherry and tumor cells expressing CLDN12‐GFP were co‐cultured and directly imaged at cell–cell junctions without permeabilization.

### 4.7 Co‐Immunoprecipitation Assay

To investigate the association between CLDN12 molecules, CAFs expressing CLDN12‐mCherry and Pan02 cells expressing CLDN12‐GFP were co‐cultured at a 1:1 ratio. Cell lysates were subjected to immunoprecipitation using anti‐GFP magnetic beads, with Protein A/G magnetic beads used as a negative control. Immunoprecipitated proteins were analyzed by immunoblotting using anti‐GFP and anti‐mCherry antibodies.

### 4.8 Immunofluorescence Staining

Orthotopic pancreatic cancer and adjacent tissues from C57BL/6 mice were collected, fixed with paraformaldehyde, embedded in paraffin, and sectioned into 6‐µm‐thick slices. After dewaxing and rehydration, the sections were stained according to the instructions of the PANO multiplex IHC 6‐color kit (PN100718AD, PANOVUE, Beijing, China). CLDN12 was labeled with PPD570, wide spectrum Cytokeratin with PPD520, and α‐SMA with PPD650. The results of immunohistochemical staining were acquired using VECTRA.

### 4.9 Collagen Gel Shrinkage Assay

Fibroblast‐mediated collagen contraction was assessed using a collagen I gel contraction assay, and gel area was quantified using ImageJ.

### 4.10 Tumor Growth and Liver Metastasis Models

Pan02‐GFP‐Luc or KPC‐Luc cells were mixed with fibroblasts at a 1:3 ratio and inoculated into C57BL/6 mice to establish tumor growth and metastasis models. Intrasplenic injection was used to establish liver metastasis, whereas orthotopic pancreatic implantation was used to evaluate primary tumor growth and spontaneous metastasis. For mechanistic studies, CLDN12‐knockdown iMEFs and corresponding control cells were used. Tumor progression and metastasis were monitored using an IVIS imaging system, and liver tissues were collected for histological analysis.

### 4.11 Treating Liver Metastasis With CLDN12‐Peptide Mimics In Vivo

After 5 days of inoculating Pan02 cells and CAFs into the spleen of mice, tumor formation was detected by IVIS, and the tumor‐bearing mice were randomly divided into different treatment groups. The CLDN12 peptide mimics (C12‐pm: YNSHLNRKFEP) and the control peptide (Con‐p: LYQY) (China Peptides, Shanghai, China) were dissolved in a 30% DMSO in water stock solution just before use, and PBS was added to bring the final concentration of DMSO to under 0.5% of the final volume of the media. Peptide (500 µg in 200 µL per mouse) was intravenously injected into mice. The injection was given once through the tail vein and repeated every other day for a total of six injections. After the end of the treatment period, all mice were euthanized after continuing observation for one week. Liver tissues were obtained, and liver metastases were detected by IVIS.

### 4.12 In Vivo Bioluminescence Imaging

Tumor metastasis was monitored using an IVIS imaging system. Mice were injected intraperitoneally with D‐luciferin potassium salt (150 mg/kg in PBS, 30 mg/mL) and anesthetized with isoflurane. Bioluminescence signals were recorded 10–15 min post‐injection to evaluate tumor burden and metastatic foci in the pancreas and liver.

### 4.13 Statistical Analysis

Unless otherwise indicated, *N* represents the number of independent biological replicates, whereas *n* represents the number of technical replicates within each biological replicate. Statistical analyses were performed using GraphPad Prism 8 (GraphPad Software Inc.). Data are represented as mean ± standard error of the mean (SEM). Comparisons between two groups were performed using Student's *t*‐test, and comparisons among multiple groups were conducted using one‐way or two‐way ANOVA. Fisher's exact test was used for categorical data. All experiments were repeated at least three times.

Detailed materials and methods can be found in the Supplementary Information.

## Author Contributions

ZQ and CN conceived and supervised the study. CN and YY conducted most of the experiments. QL constructed the expression plasmids and generated and validated stable overexpression cell lines. CN and QL analyzed the data and drafted the manuscript. ZQ, CN, and QL interpreted the data. ZQ, CN, QL, and RG critically revised the manuscript. XM primarily performed the animal studies, with assistance from RC, LZ, and XD. QF established the 3D spheroid culture system. XL contributed to cell transfection, and XY performed Western blotting. All authors read and approved the final manuscript.

## Funding

Funding was provided by National Natural Science Foundation of China [82372911 to C.N., 32370973, 82073231 to Z.Q.], Key Project of Medical Science and Technology of Henan Province [SBGJ202302037 to C.N.], Health Commission of Henan Province Outstanding Young Talent [YQRC2023005 to C.N.], and Henan Medical Researcher Overseas Training Program [HNMOT2024087 to C.N.].

## Ethic Statement

Animal experiments were approved by the Ethics Committee of the First Affiliated Hospital of Zhengzhou University (2024‐KY‐1113‐001).

## Conflicts of Interest

CN, ZQ, YY, and XD are applying a Chinese patent (202510198778.6) on the application of C12‐pm in treating pancreatic metastasis. No further conflicts of interest needs to be declared.

## Supporting information




**Supporting File 1**: mco271114‐sup‐0001‐SuppMat.docx

## Data Availability

All raw data and materials are available from the corresponding authors upon request. The public datasets analyzed in this study are GSE71729 and GSE154778.
