# Supplementary material for: Claudin‐12‐Expressing Fibroblasts Facilitate Metastatic Spread of Pancreatic Cancer Through Physical Interactions
Source: MedComm (2020). 2026 Oct 31;7(11):e71114. doi: 10.1002/mco2.71114 (PMC13722954; doi:10.1002/mco2.71114)
Supplement: Supplementary file 1 [file MCO2-7-e71114-s001.docx]

**Claudin-12-expressing fibroblasts facilitate metastatic spread of pancreatic cancer through physical interactions**

**Qingcui Li^1,2#^, Xiangjing Miao^1#^, Yuanyuan Yang^1#^, Ruoyang Chen^1^, Qingqing Fang^1^, Xixi Duan^1^, Xiaohan Lou^1^, Xiaohan Yao^1^, Lijing Zhang^1^, Rainer Glauben^3^, Zhihai Qin^1^***, **Chen Ni^1#^***

**Supplementary Methods**

**Cell lines and cell culture**

Mouse CAFs were established from fresh PDAC tissues from a KPC genetically engineered mouse model using an enzymatic digestion method. Briefly, sterile fresh surgical tissue was washed 2-3 times with 1x phosphate buffered saline (PBS, Hyclone) to remove blood contamination. The tissue was cut thoroughly with ophthalmic scissors and placed on a shaker at 37 ℃ with digestive fluid for 1 h. Digestion was terminated with DMEM containing FBS. It was then filtered through a 70 μm nylon mesh (ABC60070, ABC biochemistry Hong Kong) and centrifuged at 2, 000 g, 4ºC for 5 min. Cells were cultured in DMEM supplemented with 10% FBS and 1% penicillin-streptomycin. After 2-3 days, fibroblasts were purified by magnetic bead sorting using Anti-FITC MicroBeads (Miltenyi Biotec, 130-048-701) following staining with FITC-conjugated anti-PDPN.

**Western blotting**

Cells were lysed with RIPA (R0020, Solarbio, China) lysis buffer on ice for 30 minutes. Protein concentrations were determined with a Pierce^TM^ BCA Protein assay kit (23228, Thermo Scientific). After separation by SDS-PAGE, proteins were transferred onto nitrocellulose (NC) membranes (pore size 0.45 µm, Merck Millipore, Ireland) and incubated with primary antibodies overnight at 4°C. The following day, blots were incubated in horseradish-peroxidase-conjugated (HRP) secondary antibody diluted in 5% milk TBST for 1 h at room temperature. The primary antibodies included CLDN12 (18801, IBL, 1:200), β-actin (AC026, ABclonal, 1:5 000), α-SMA (19245s, CST, 1:1 000), COL1A1 (HA722517, HUABIO, 1:1 000), S100A4 (ab41532, Abcam, 1:1 000), SMAD2 (5339S, CST, 1:1 000), p-SMAD2 (3108S, CST,1:1 000).

**Dot Blot Assay**

Conditioned media from Pan02 cells collected at 24 h and 48 h were filtered through a 0.22 µm membrane and concentrated using 10-kDa ultrafiltration tubes to equal volumes. Two microliters of each concentrated sample were spotted onto pre-wetted nitrocellulose (NC) membranes and air-dried at room temperature for approximately 2 h. The membranes were then blocked with 5% non-fat milk in TBST for 1 h at room temperature, followed by incubation with an anti–TGF-β primary antibody at 4 °C overnight and an HRP-conjugated secondary antibody for 1 h at room temperature. After thorough washing with TBST, signals were developed using an ECL detection reagent.

**RNA extraction and qRT-PCR**

FastPure Cell/Tissue Total RNA Isolation Kit V2 (RC112-01, Vazyme, Nanjing, China) was used to extract total RNA. The reverse transcription reaction was carried out using a reverse transcription kit (RR036A, Takara, China). All procedures were performed according to the manufacturer’s instructions. The SYBR Premix Ex Taq kit (RR820A, Takara, China) was used to quantify the expression of CLDN12 mRNA on the Bio-Rad CFX384 Real-Time 384-well PCR detection system. The following primer pairs were used (forward/reverse; 5′-3′): mouse **CLDN12** (Cldn12) (GGACTCTGCTTCCTAACTGGAG / TCAGGCAGTCACTGCTTCCATC), mouse **18S rRNA** (ACCGCAGCTAGGAATAATGGA / CAAATGCTTTCGCTCTGGTC), **α-SMA** (Acta2) (GGCACCACTGAACCCTAAGG / ACAATACCAGTTGTACGTCCAGA), **COL1A1** (Col1a1) (TAAGGGTCCCCAATGGTGAGA / GGGTCCCTCGACTCCTACAT), **Fibronectin** (Fn1) (CGCAAAAGCAAAAGGAAGAC / CAGGTCTTGGGAGTGGTTGT), and **Podoplanin** (Pdpn) (GTGTAACAGGCATTCGCATCG / TGTGGCGCTTGGACTTTGT). Relative mRNA expression was calculated using the 2^−ΔΔCt^ method, with 18S rRNA as the reference gene. The experiments were repeated at least three times.

**Lentiviral shRNA transduction**

Lentiviral vectors encoding CLDN12-targeting shRNAs or a non-targeting control shRNA were constructed by Genechem (Shanghai, China) using an hU6-MCS-Ubiquitin-EGFP-IRES-puromycin backbone. Lentiviral particles were produced by transfecting HEK293T cells with the indicated shRNA plasmids using jetPRIME transfection reagent (101000046, Polyplus, Shanghai, China). Twenty-four hours later, the medium was changed to fresh DMEM with 10% FBS. The supernatant was collected every 24 hours and filtered with 0.45 μm nitrocellulose filter. Supernatants were used to infect fibroblasts and Pan02 cells for 24 h and then selected with 2 μg/mL puromycin. The stable pooled clones were verified by qRT-PCR or Western blotting.

**Plasmid transfection for CLDN12 overexpression**

For CLDN12 overexpression, fibroblasts or Pan02 cells were transfected with plasmids encoding mouse CLDN12 fused at the C terminus with GFP or mCherry (CLDN12-GFP or CLDN12-mCherry) using Lipofectamine 2000 (Invitrogen) according to the manufacturer’s instructions. Fluorescent protein-positive cells were subsequently enriched by fluorescence-activated cell sorting (FACS). CLDN12 overexpression was confirmed by Western blotting.

**Wound-healing migration assay**

For the wound-healing assay, three horizontal reference lines were drawn on the underside of 96-well plates. Pan02^sh-nc^ cells (n = 6) and Pan02^sh^*^Cldn12^* cells (sh2, sh3; n = 6) were seeded and cultured overnight. A vertical scratch was made across the center of each well using a pipette tip held perpendicular to the plate, ensuring the scratch intersected the reference lines. After removing the medium, wells were washed three times with PBS to eliminate detached cells. Images were taken immediately (0 h) using a Leica microscope. Wound width was monitored for 24 h, and relative wound width was quantified using ImageJ.

**H&E Staining**

Liver tissues were harvested from tumor-bearing mice, fixed in 4% paraformaldehyde at room temperature for 24 h, and embedded in paraffin. Sections (4-5 µm) were cut, deparaffinized in xylene, and rehydrated through a graded ethanol series. Sections were then stained with hematoxylin for 5-10 min, rinsed in running tap water, differentiated in 1% acid alcohol if necessary, and counterstained with eosin for 2-5 min. After dehydration through ascending ethanol concentrations and clearing in xylene, sections were mounted with neutral balsam. Stained sections were imaged using a light microscope (Leica, Germany) for histopathological analysis of liver metastases.

**TGF-β signaling inhibition**

Pan02 cells were cultured in complete DMEM until approximately 80% confluence. For conditioned medium (CM) collection, cells were washed with PBS and incubated in serum-free DMEM for 24 h. The collected supernatants were centrifuged to remove cell debris and used as Pan02-conditioned medium (Pan02-CM). iMEF cells were treated with Pan02-CM for the indicated time periods to evaluate changes in CLDN12 expression.

For TGF-β receptor inhibition experiments, iMEF cells were treated with the TGF-β receptor inhibitor SD208 (2 μM) in the presence of recombinant TGF-β or Pan02-CM for 24 h. For adhesion assays, iMEFs were treated with TGF-β or Pan02-CM with or without SD208, followed by co-culture with Pan02-GFP-Luc cells. Cancer cell-fibroblast adhesion was quantified using the flow cytometry-based adhesion assay described above.

**Cancer cell-fibroblast adhesion assay**

Cancer cell-fibroblast adhesion was evaluated using two complementary approaches, including a plate-based adhesion assay (Figure 3A) and a flow cytometry-based tube adhesion assay (Figure 3H). For plate-based adhesion, pancreatic cancer cells (1×10⁵ cells/well) were seeded into 12-well plates and cultured overnight. GFP-labeled fibroblasts (1×10⁵ cells/well) were then added onto the cancer cell monolayer, followed by centrifugation at 100 g for 5 min to promote initial contact. After incubation at 37°C and 5% CO₂ for 15 min, non-adherent cells were removed by washing with PBS three times. After washing, the remaining GFP-positive fibroblasts attached to the cancer cell monolayer were considered adherent cells and quantified by fluorescence microscopy.

For the tube-based adhesion assay, CAFs were labeled with CellTrace Violet (Thermo Fisher), and cancer cells were labeled with PKH26 (Maokang Bio) according to the manufacturers’ instructions. Equal numbers of labeled fibroblasts and cancer cells were mixed, centrifuged at 200 g for 4 min, and co-incubated for 20 min at 37°C. Cell-cell adhesion was quantified as the percentage of double-positive (Violet⁺PKH26⁺) events. Double-positive events represent fibroblast–cancer cell conjugates.

**Scratch-Invasion assay**

Three horizontal reference lines were drawn on the underside of 96-well plates. GFP-labeled fibroblasts and mCherry-labeled tumor cells were co-seeded at a 2:1 ratio and cultured overnight. A vertical scratch was generated through the center of each well using a pipette tip held perpendicular to the plate, ensuring intersection with the reference lines. Wells were washed three times with PBS to remove detached cells, and baseline images were acquired at 0 h (Leica, Germany). Immediately after scratching, 40 µL Matrigel (2.5 mg/mL; 354234, Corning, USA) was added to each well and allowed to solidify at 37 °C for 1 h. Cells were then cultured for an additional 24 h to assess invasion into the Matrigel. For each experimental condition, images were collected from five fields per well across eight wells. Relative wound width and invasion distance were quantified using ImageJ.

**Spheroid 3D Invasion Assay**

CAFs (4,000 cells) and Pan02 cells (2,000 cells) were mixed and resuspended in 20 µL DMEM containing 0.24% (w/v) methylcellulose to form tumor cell-fibroblast co-spheroids using the hanging drop method. Spheroids were incubated overnight at 37°C to allow spheroid formation. The next day, 50 µL Matrigel diluted 1:1 with DMEM was added to the bottom of each well in a 96-well plate. Spheroids were gently collected, embedded in Matrigel, and incubated at 37°C for 30 min to allow gelation. Subsequently, 100 µL complete DMEM was added, and spheroids were cultured for 24 h to induce invasion. Spheroid invasion was imaged using an UltraVIEW VoX confocal microscope (PerkinElmer, USA). The invasion area extending from the spheroid was quantified using ImageJ software (N = 5 independent spheroids per group).

**Transwell migration assay**

For coculture migration assays, 5 × 10⁴ GFP-labeled fibroblasts and 5 × 10⁴ mCherry-labeled pancreatic cancer cells were seeded in the upper chamber of 8-μm pore Transwell inserts (3422, Corning, USA) in serum-free DMEM. DMEM containing 10% FBS was added to the lower chamber as a chemoattractant. After 24 h, Pan02 cells were identified by their fluorescent label and imaged using a fluorescence microscope. Image fields were randomly selected, and cell numbers were quantified with ImageJ. For single-cell migration assays, 1 × 10⁵ Pan02^sh-nc^ or Pan02^sh^*^Cldn12^* cells (sh2, sh3) were seeded in the upper chamber under the same conditions. Migrated cells were fixed and stained with 0.1% crystal violet for 30 min before counting, and cell numbers were quantified using ImageJ. At least three independent biological replicates were performed, with 3-9 randomly selected fields captured per group.

**Cellular immunofluorescence**

Cells were seeded on confocal glass coverslips and cultured at 37 °C in 5% CO₂ until approximately 80% confluence. For immunostaining of intracellular markers, cells were washed three times with PBS and fixed with 4% paraformaldehyde at room temperature for 10 min, followed by permeabilization with 0.1% Triton X-100 for 10 min. After blocking with 3% fetal bovine serum for 30 minutes, samples were incubated overnight at 4 °C with primary antibodies, including rabbit anti-CLDN12 (IBL, 1:20) or anti-α-SMA, washed with PBS, and then incubated with appropriate Alexa Fluor-conjugated secondary antibodies (1:200) for 1 h at 37 °C. Nuclei were counterstained with DAPI for 10 min, washed, and imaged using laser-scanning confocal microscopy. For visualization of CLDN12-mediated cell-cell interactions, fibroblasts expressing CLDN12-mCherry and tumor cells expressing CLDN12-GFP were co-cultured on coverslips. Cells were fixed with 4% paraformaldehyde without permeabilization, and GFP/mCherry fluorescence at cell-cell junctions was directly imaged by confocal microscopy.

**Co-immunoprecipitation assay**

CAFs expressing CLDN12-mCherry and Pan02 cells expressing CLDN12-GFP were co-cultured at a 1:1 ratio for 24 h. Cells were lysed in IP lysis buffer (NP-40 Lysis Buffer, Beyotime, Cat# P0013F) supplemented with protease inhibitor cocktail (Beyotime, Cat# P1046). Cell lysates were centrifuged at 12,000 rpm for 10 min at 4°C to remove insoluble debris. Equal amounts of total protein (500 μg per immunoprecipitation) were incubated with Anti-GFP Magnetic Agarose Beads (MCE, Cat# HY-K0246, 40 μL) or BeyoMag™ Rabbit IgG Magnetic Beads (Beyotime, Cat# P2173, 20 μL) at 4°C for 1-2 h to capture GFP-associated protein complexes. The beads were washed 3–5 times with TBST, and bound proteins were eluted with SDS sample buffer. Immunoprecipitated proteins were separated by SDS-PAGE and subjected to immunoblotting using anti-mCherry (Proteintech, Cat# 26765-1-AP, 1:1000) and anti-GFP (Proteintech, Cat# 50430-2-AP, 1:1000) antibodies.

**Time-Lapse Microscopy**

CAFs (CAF^sh-nc^ or CAF^sh^*^Cldn12^*) and Pan02 tumor cells were fluorescently labeled (GFP for CAFs and mCherry for Pan02 cells). Labeled cells were co-embedded in Matrigel-coated confocal dishes at a 1:1 ratio and incubated at 37°C with 5% CO₂. Time-lapse imaging was performed using a confocal microscope (UltraVIEW VoX, PerkinElmer), with images captured every 1 h for 3 h. Cancer cells physically associated with fibroblasts were tracked using ImageJ (Manual Tracking plugin), and cell motility speed was calculated. At least three independent experiments were performed, with ≥20 cell pairs analyzed per condition.

**Collagen gel shrinkage assay**

Collagen (1 mg/mL, 354249., Corning, USA) was mixed with 0.1 mol/L NaOH, and appropriate volume of PBS to adjust the pH to approximately 7. A pre-calculated volume of cell suspension was then added and mixed thoroughly. The cell-collagen mixture was dispensed into 96-well plates and allowed to polymerize at room temperature for 30 min. Subsequently, 100 μL DMEM was added to each well, and the plates incubated at 37ºC with 5% CO2. After 24 h, the medium was replaced with complete medium with or without 10 ng/mL TGF-β and cells were incubated for an additional 48 h. Images were taken with a digital camera and the changes in collagen gel area were calculated using ImageJ. The percentage of gel shrinkage was calculated using the formula: [(initial gel area - area after contraction) / initial area] × 100%.

**Tumor Growth and Liver Metastasis Models**
Pancreatic cancer cells (Pan02-GFP-Luc or KPC-Luc, 5 × 10⁵) were mixed with fibroblasts (iMEFs, 1.5 × 10⁶) at a 1:3 ratio in DMEM and Matrigel (1:1, v/v).

Two metastasis models were established:
(1) **Intrasplenic injection model**: cells were injected into the spleen of anesthetized C57BL/6 mice to assess liver metastasis.
(2) **Orthotopic pancreatic implantation model**: cells were injected into the pancreas to evaluate primary tumor growth and spontaneous metastasis.

For mechanistic studies, iMEF cells with CLDN12 knockdown (iMEF^sh^*^Cldn12^*) and their negative controls (iMEF^sh-nc^) were used. Tumor metastasis was monitored using the IVIS imaging system. At the endpoint, liver tissues were collected, fixed in 4% paraformaldehyde, embedded in paraffin, sectioned, and stained with H&E.

**Analysis of public human PDAC datasets**

Public human PDAC datasets were analyzed to assess the clinical relevance of CLDN12. Bulk transcriptomic data from GSE71729 were used to compare CLDN12 expression among normal pancreas, primary PDAC, and metastatic PDAC. The Kruskal-Wallis test was used to assess overall differences among the three groups, and pairwise comparisons were performed using two-sided Wilcoxon rank-sum tests with Benjamini-Hochberg (BH) correction. Single-cell RNA-seq data from GSE154778 were analyzed using Seurat. Cells were clustered and annotated into major cell types, and CLDN12 expression was visualized on UMAP plots.

**Supplementary Figures and Figure legends**

**Supplementary Figure S1. Pan02-conditioned medium induces fibroblast activation and CLDN12 promotes malignant phenotypes of pancreatic cancer cells *in vitro*.** (**A**) Western blot analysis of α-SMA expression in iMEFs treated with or without Pan02-conditioned medium (CM), β-actin was used as a loading control. (**B**) Western blot analysis of CLDN12 protein expression in KPC and Pan02 cells. (**C**) qRT-PCR analysis of *Cldn12* mRNA expression in MEF and Pan02 cells (N = 3, *t*-test). (**D**) The expression of *Cldn12* mRNA in Pan02 transfected with sh-nc and sh*Cldn12* (sh2, sh3) (N = 3, one-way ANOVA). (**E**) The proliferation of Pan02 cells transfected with sh-nc and sh*Cldn12* (sh2, sh3) detected by CCK-8 (N = 3, two-way ANOVA). (**F, G**) Clone formation assay was performed with Pan02^sh-nc^ and Pan02^sh^*^Cldn12^* cells (n = 6, *t*-test). (**H, I**) The migration of Pan02^sh-nc^ (N = 3) and Pan02^sh^*^Cldn12^* cells (sh2, sh3) (N = 3) detected with Transwell assay. One-way ANOVA. (**J, K**) The migration of Pan02^sh-nc^ (n = 6) and Pan02^sh^*^Cldn12^* cells (sh2, sh3) (n = 6) detected with wound healing assay. One-way ANOVA.

**Supplementary** **Figure S2. CLDN12 knockdown in CAFs reduces adhesion to pancreatic cancer cells.** (**A, B**) The adhesion of iMEF^sh-nc^ and iMEF^sh^*^Cldn12^* cells to KPC-Luc cells detected by flow cytometry. N = 6 for each group, *t*-test. (**C**) The expression of *Cldn12* mRNA in MEF cells isolated from *Cldn12*^+/+^ or *Cldn12*^−/−^ mice. N = 3 for each group, *t*-test. (**D, E**) Flow cytometric analysis of the adhesion between Pan02-GFP-Luc cells and CAF^sh-nc^, CAF^sh^*^Cldn12^* (sh2, sh3). N = 6 for each group, one-way ANOVA. (**F**) Flow cytometry gating strategy for the cancer cell-fibroblast adhesion assay. CellTrace Violet⁺PKH26⁺ double-positive populations were defined as tumor cell-fibroblast adhesion events. (**G**) Western blot analysis of CLDN12 overexpression in CAFs. The left lane represents the control group, and the right lane represents the CLDN12-overexpressing group. Cell lysates were analyzed by immunoblotting with an anti-CLDN12 antibody, and β-actin was used as a loading control. Densitometric analysis of the CLDN12/β-actin ratio is shown.

**Supplementary** **Figure S3. Screening of cytokines regulating CLDN12 expression and effects of exogenous TGF-β on CLDN12 expression and cancer cell-fibroblast adhesion.** (A-D) iMEF cells were treated with TNF-α (**A**), IFN-γ (**B**), S100A4 (**C**), or GM-CSF (**D**) for 24 h, and *Cldn12* mRNA expression was measured by qPCR. N = 6 for each group, *t-*test. (**E**) iMEF cells were treated with TGF-β (5 ng/mL, 10 ng/mL) for 24 h, and CLDN12 protein expression was analyzed by Western blotting. (**F-G**) iMEF cells were treated with recombinant TGF-β with or without SD208, followed by co-culture with Pan02-GFP-Luc cells. Cancer cell-fibroblast adhesion was analyzed by flow cytometry-based adhesion assay. N = 6 for each group, one-way ANOVA.

**Supplementary** **Figure S4.** **Effects of CLDN12-expressing fibroblasts on tumor growth in subcutaneous and orthotopic pancreatic tumor models.**

(**A**) Schematic illustration of subcutaneous (s.c.) co-inoculation of Pan02-GFP-Luc cells with iMEF cells in wild-type C57BL/6 mice. (**B**) Tumor growth curves for co-injection of Pan02-GFP-Luc with iMEF cells. n = 5 for each group, two-way ANOVA. (**C**) Images showing Pan02 tumors at day 32 post-inoculation. (**D**) Representative ex vivo bioluminescence images of primary tumors from an orthotopic pancreatic implantation model in mice co-implanted with Pan02-GFP-Luc cells and iMEF^sh-nc^ or iMEF^sh^*^Cldn12^* fibroblasts. (**E**) Quantification of ex vivo bioluminescence signal intensity of orthotopic primary tumors in the indicated groups. The iMEF^sh-nc^ group included 13 mice, and the iMEF^sh^*^Cldn12^* group included 12 mice. Statistical analysis was performed using t-test. (**F**) Representative ex vivo bioluminescence images showing liver metastasis in mice from the orthotopic pancreatic tumor model co-implanted with Pan02-GFP-Luc cells and iMEF^sh-nc^ or iMEF^sh-Cldn12^ fibroblasts. Quantification of liver metastatic burden is shown in Figure 6D. (**G**) Representative ex vivo bioluminescence images showing colon metastasis in mice from the orthotopic pancreatic tumor model. (**H**) Incidence of colon metastasis in the indicated groups. The iMEF^sh-nc^ group included 13 mice, and the iMEF^sh^*^Cldn12^* group included 12 mice. Statistical analysis was performed using Fisher’s exact test.

**Supplementary** **Figure S5. CLDN12 peptide mimics (C12-pm) inhibit fibroblast-mediated adhesion, including TGF-β-activated CAFs.** (**A**) Representative fluorescence images showing GFP-expressing iMEF cells adhering to Pan02 monolayers after pretreatment with CLDN12 peptide mimics (C12-pm) or control peptide (Con-p) for 24 h. Scale bar: 200 μm. (**B**) Quantification of adherent GFP-positive iMEF cells shown in (A)*.* N = 3, one-way ANOVA. (**C**) Adhesion of Pan02-GFP-Luc cells to TGF-β–stimulated (10 ng/mL) CAFs pretreated with Con-p or C12-pm (400 μM) for 24 h.

**Supplementary Figure S6**. **CLDN12 expression in human PDAC datasets**.

**(A)** Violin plots showing CLDN12 expression in normal pancreas, primary PDAC, and metastatic PDAC from GSE71729. Each black dot represents one sample. Black horizontal lines indicate the median. The y-axis indicates log2-normalized CLDN12 expression. The Kruskal-Wallis test showed a significant difference among the three groups (p = 3.84e-12), and pairwise comparisons were performed using two-sided Wilcoxon rank-sum tests with Benjamini-Hochberg (BH) correction (all BH-adjusted p < 0.001). n = 134 for normal, n = 145 for primary PDAC, and n = 61 for metastatic PDAC.

**(B)** UMAP plot of major cell types in human PDAC from GSE154778. umap_1 and umap_2 indicate UMAP dimensions. **(C)** UMAP plot showing CLDN12 expression across the major cell types in (B). Color scale indicates log-normalized CLDN12 expression, ranging from 1.00 to 2.00. CAFs showed detectable but relatively low CLDN12 expression. The red ovals in (B) and (C) highlight the CAF cluster.
